# Health-related quality of life during the COVID-19 pandemic: The impact of restrictive measures using data from two Dutch population-based cohort studies

**DOI:** 10.1371/journal.pone.0300324

**Published:** 2024-03-18

**Authors:** Cheyenne C. E. van Hagen, Anne J. Huiberts, Elizabeth N. Mutubuki, Hester E. de Melker, Eric R. A. Vos, Janneke H. H. M. van de Wijgert, Susan van den Hof, Mirjam J. Knol, Albert Jan van Hoek

**Affiliations:** 1 Centre for Infectious Disease Control, National Institute for Public Health and Environment (RIVM), Bilthoven, the Netherlands; 2 Julius Center for Health Sciences and Primary Care, University Medical Center Utrecht (UMCU), Utrecht, the Netherlands; Instituto Nacional de Salud Pública: Instituto Nacional de Salud Publica, MEXICO

## Abstract

**Objectives:**

We describe health-related quality of life during the COVID-19 pandemic in the general Dutch population and correlations with restrictive measures.

**Methods:**

Data were obtained from 18–85 year-old participants of two population-based cohort studies (February 2021-July 2022): PIENTER Corona (n = 8,019) and VASCO (n = 45,413). Per cohort, mean scores of mental and physical health and health utility from the SF-12 were calculated by age group, sex and presence of a medical risk condition. Spearman correlations with stringency of measures were calculated.

**Results:**

Both cohorts showed comparable results. Participants <30 years had lowest health utility and mental health score, and highest physical health score. Health utility and mental health score increased with age (up to 79 years), while physical health score decreased with age. Women and participants with a medical risk condition scored lower than their counterparts. Fluctuations were small over time but most pronounced among participants <60 years, and correlated weakly, but mostly positively with measure stringency.

**Conclusions:**

During the Dutch COVID-19 epidemic, health utility and mental health scores were lower and fluctuated strongest among young adults compared to older adults. In our study population, age, sex and presence of a medical risk condition seemed to have more impact on health scores than stringency of COVID-19 non-pharmaceutical interventions.

## Introduction

The coronavirus disease 2019 (COVID-19) outbreak, caused by severe acute respiratory syndrome coronavirus 2 (SARS-CoV-2), was declared a pandemic by the World Health Organization on 11 March 2020 [1]. To curb the spread of the virus, governments worldwide implemented various strategies ranging from limited to highly restrictive measures. The Netherlands adopted general measures, including physical distancing and hygiene measures, but also more restrictive measures like closure of schools, home confinement, social distancing, and a curfew.

It is expected that the COVID-19 pandemic impacted health-related quality of life (HRQoL) of all people in the population, regardless of infection. Published studies from the early stages of the COVID-19 pandemic reported increased rates of psychological distress, such as anxiety, and depression [2–4]. Quarantine as public health intervention was found to have had a negative psychological impact [5]. Next to the pandemics’ psychological impact, productivity loss, postponed or cancelled care and decreased physical activity levels were also experienced during the COVID-19 pandemic [4, 6, 7]. Age-related differences on mental and physical health have been reported, whereby the mental health of younger people was affected more, also illustrated by an increased prevalence of suicidal thoughts [8, 9]. Youth more often suffered from depressive symptoms, anxiety and loneliness, and also ate less healthy and exercised less frequently [9]. The extent of the impact of the COVID-19 pandemic on health utility, mental and physical health over time, in population subgroups and whether the impact was correlated with the stringency of restrictive measures remains unknown.

In this study we primarily aimed to describe the HRQoL, stratified by age, sex and presence of a medical risk condition in the general population in the Netherlands, during the second and third year of the COVID-19 pandemic using observational data from two large prospective cohorts. The secondary objective was to investigate whether fluctuations in HRQoL over time correlated with the stringency of restrictive measures.

## Materials and methods

### Study design and population

Data from two different population-based prospective cohort studies in the Netherlands were used: VAccination Study COrona (VASCO) [10] and PIENTER Corona (PICO) [11, 12]. Both cohorts were sampled from the general population and asked to complete the Dutch translation of the 12-item Short-Form Health Survey (SF-12) version 1. We analysed data from both cohorts to improve robustness and validity of our results and to cover a longer observation period. Participants from both cohorts aged between 18 and 85 years, and who completed the SF-12 questionnaire at least once, were included. Both studies were conducted in accordance with the principles of the Declaration of Helsinki. The VASCO study protocol was approved by the not-for-profit independent Medical Ethics Committee of the *Stichting Beoordeling Ethiek Biomedisch Onderzoek* (BEBO), Assen, the Netherlands (Clinical Trial Registration NL9279). The PICO study protocol was approved by the Medical Ethics Committee MEC-U, the Netherlands (Clinical Trial Registration NTR8473). All participants provided written informed consent.

VASCO is an ongoing population-based prospective cohort study, in which 45,553 community-dwelling persons aged 18–85 years were enrolled between 3 May 2021 and 15 December 2021 [10]. Participants were recruited through random sampling of the Dutch population register and campaigns on social media and traditional media. Participants had to be able to understand Dutch, as all study materials were written in Dutch, and were included irrespective of their COVID-19 vaccination status or intention to get vaccinated. The main aim of this 5-year follow-up study is to estimate vaccine effectiveness of COVID-19 vaccination against SARS-CoV-2 infection over time. At baseline, data on sociodemographic variables and health status were collected. Next to questions regarding SARS-CoV-2 testing and COVID-19 vaccination used for estimating vaccine effectiveness, participants were asked to complete the SF-12 questionnaire to assess their HRQoL at baseline and every three months. Since participants entered the VASCO cohort over a period of 6 months and the SF-12 questionnaire is administered every three months, continuous data are available from 3 May 2021 until 31 July 2022.

PICO is a nationwide, population-based seroepidemiological cohort study investigating SARS-CoV-2 antibodies and risk factors among the Dutch population as its primary aim. Participants were initially sampled in March 2020 from the in 2016/2017 established PIENTER-3 serosurvey cohort in which participants were invited via a two-stage cluster design [11, 12]. To enhance countrywide geographical coverage and maintain power, the PICO study population was supplemented twice (in June 2020 and November 2021) by actively inviting additional persons randomly selected from the Dutch population registry [13]. This resulted in an overall study population of 11,159 participants aged 1–92 years. In each round, participants completed a questionnaire including questions on sociodemographic factors, health status, COVID-19 vaccination, and SARS-CoV-2-related symptoms. Study rounds from February 2021 onwards additionally included the SF-12 questionnaire to assess HRQoL for participants aged 15 years and older. To keep study periods more concise, data of the first four weeks of each round (February, June, and November 2021) were included, covering the majority of the completed questionnaires (98.4%, 94.5% and 95.4%, respectively).

### Outcome measures

The SF-12, an abbreviated version of the SF-36 HRQoL questionnaire, includes 12 items covering eight health concepts: physical functioning (PF), role limitations due to physical health problems (RP), bodily pain (BP), general health (GH), vitality (energy/fatigue) (VT), social functioning (SF), role limitations due to emotional problems (RE) and mental health (psychological distress and psychological wellbeing) (MH) [14]. These concepts are converted into a physical health (PCS) and mental health (MCS) component score. The PCS score primarily encompasses answers to PF, RP, BP and GH questions and the MCS score answers to VT, SF, RE and MH questions. PCS and MCS scores were weighted using standard regression coefficients obtained in the general US population [15, 16]. PCS and MCS scores range from 0 to 100 with higher scores denoting better health and a score of 50 indicating average health. Minimal clinically important differences (MCID) for PCS and MCS scores are suggested to range between 3 and 5 [17, 18], but may be somewhat outside this range in certain patient groups [19–22]. In addition to PCS and MCS scores, Short-Form Six Dimensions (SF-6D) health utility scores were derived from seven items from the SF-12 covering six of its health concepts (PF, role limitations (RE/RP), SF, BP, MH and VT) [23]. Scores range from 0.3 to 1 (UK population average 0.8), with 1 indicating full health [23, 24]. The MCID of the SF-6D is estimated to be 0.033 (95% CI: 0.029 to 0.037) with a range of 0.010 to 0.048 [25].

The SF-12 version 1 measured HRQoL related to mental health, physical health and health utility in the four weeks prior to completion. In VASCO and the online PICO questionnaire in the February 2021 round, participants received the SF-12 version 1 UK. The paper-based February 2021 questionnaire and all questionnaires of subsequent PICO rounds contained the SF-12 version 1 US. Results of the SF-12 version 1 UK were transformed to the SF-12 version 1 US following standard procedure and all questionnaires were analysed in the SF-12 version 1 US format [26, 27].

### Covariates

As a proxy for the stringency of COVID-19 measures in the Netherlands, the Dutch Stringency Index of the Oxford Coronavirus Government Response Tracker (OxCGRT) was used [28]. The Stringency Index is a composite measure based on nine response indicators such as school closures, workplace closures, and travel bans, rescaled to a value from 0 to 100 (strictest), which records the strictness of government policies [28].

Four risk groups were defined combining age group (18–59 and 60–85 years) and the presence of a medical risk condition (yes/no). A medical risk condition was defined as reporting at least one of the following physician-diagnosed and/or drug-treated conditions: asthma, diabetes (type 1, type 2 and type unknown), asplenia, cardiovascular disease, immune deficiency, cancer (currently, treated and currently, untreated), liver disease, lung disease, neurological disease, kidney disease and organ or bone marrow transplantation.

### Study period

**Fig 1:**
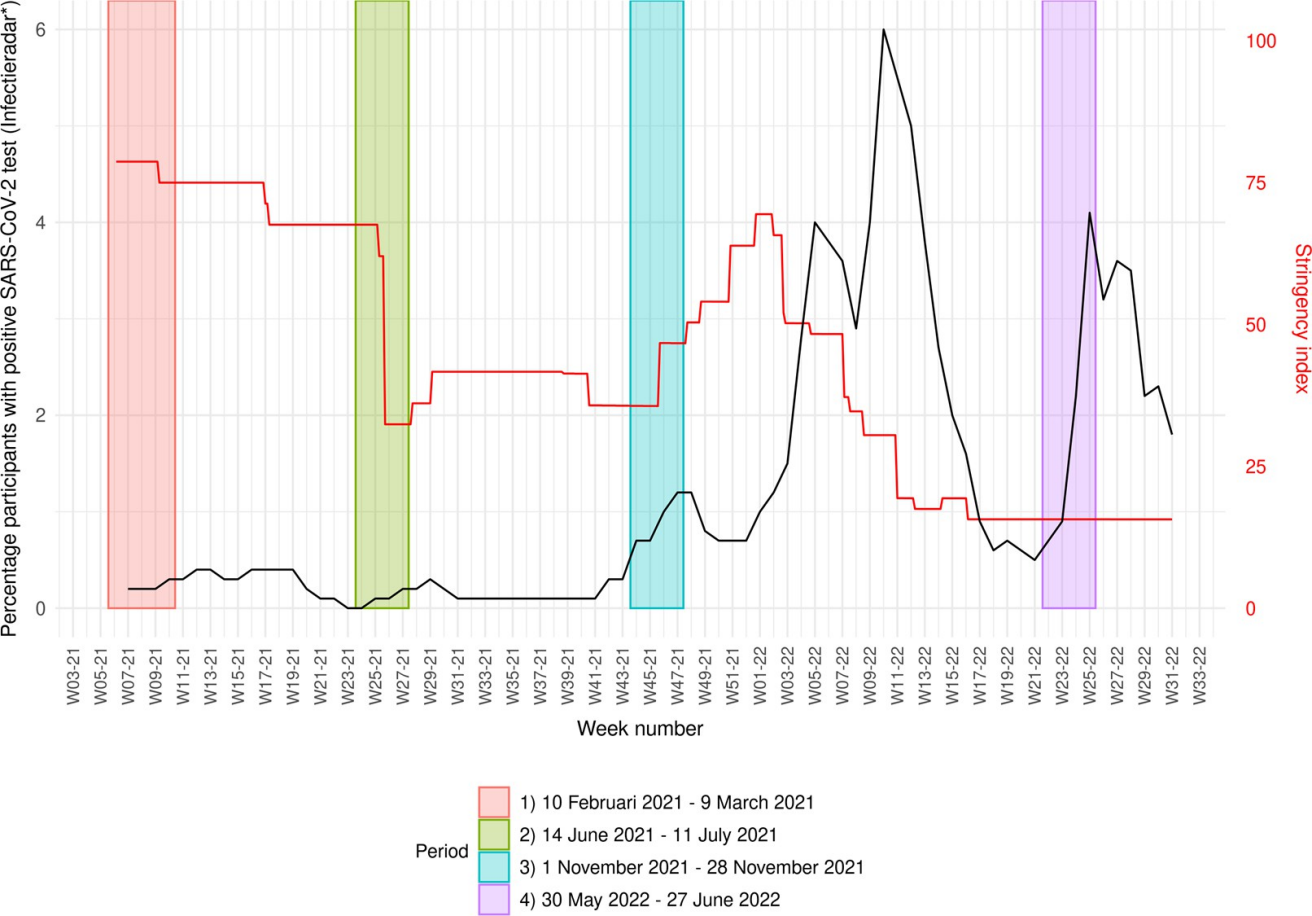
Timeline of OxCGRT’s Stringency Index and SARS-CoV-2 infection incidence* in the Netherlands during our study period with four predefined and separately studied periods colour-indicated. * Dutch incidence data (Infectieradar) is available from: https://www.infectieradar.nl/results.

Data between 10 February 2021 and 31 July 2022 were available from the two cohorts, and were analysed separately. A complete case analysis was performed. We defined three four-week time periods based on PICO study rounds in 2021 and an additional four-week period with few restrictive measures in 2022: (1) 10-02-2021–09-03-2021; (2) 14-06-2021–11-07-2021; (3) 01-11-2021–28-11-2021; (4) 30-05-2022–27-06-2022. The four time periods captured different stages of the epidemic curve as well as different phases of restrictive measures that were in place (**Fig 1**). In period one, incidence of infections was relatively low, but there was a strict lockdown including curfew and closure of non-essential shops. Incidence was low in period two, but the Delta variant of concern caused a peak in infections in period three. In both periods, restrictive measures were less intensive than in period one, with intensiveness decreasing in period two and increasing in period three. Period four captures a period in which there is a peak in infections caused by the Omicron BA.5 variant of concern (i.e., the third Omicron wave in the Netherlands), but most restrictive measures were lifted.

### Statistical analysis

Characteristics of the participants in both cohorts were presented using descriptive statistics to assess distribution of the prognostic factors age, sex, ethnic background, comorbidities, risk groups and educational level.

Primary objective: Mean PCS, MCS and SF-6D utility scores with standard deviations (SDs) during each of the four different periods were calculated cross-sectionally as well as averages over the four periods. Results were stratified by age, sex and medical risk condition. Per age group, scores between males and females and those with and without a medical risk condition were tested for significance using a two-sample t-test. P-values <0.05 were considered statistically significant. Stratification by sex excluded participants who classified their sex as other, as limited numbers were available. Additionally, distributions of scores of each SF-12 item were studied separately by age group.

Secondary objective: Mean weekly PCS, MCS and SF-6D utility scores with 95% confidence interval (CI) were calculated for VASCO participants over the period of 17 May 2021 to 31 July 2022. Questionnaires that were completed between 3 and 17 May 2021 (n = 5) were excluded because of limited inclusion numbers in these first two weeks. Results were stratified by risk group. PCS, MCS and SF-6D utility scores were modelled as a function of risk group and time using a generalized additive model from the R package mgcv (with penalized B-splines, 20 knots). For each outcome, Spearman correlations with the OxCGRT’s Stringency Index were calculated, with a lag time of two weeks representing the midpoint of the four weeks measured in the SF-12.

Multiple sensitivity analyses were performed. Firstly, all analyses were repeated using Dutch population weights [29] for PCS and MCS and crude sum scores of SF-12 items associated with mental or physical health [30, 31]. Secondly, for our primary objective we weighted the results of both cohorts to the general Dutch population based on age, sex, ethnicity and area of residence to ensure comparability between the two cohorts. Lastly, for our secondary objective, Spearman correlations were calculated using varying lag times of zero, one, three and four weeks. Additionally, we explored correlations for the pre-Omicron period (until 27 December 2021) and Omicron period (from 27 December 2021) with two-week lag-time separately, as the Omicron period marked the start of a new phase in the pandemic with high infection numbers but decreased individual disease burden and less restrictive measures.

Data cleaning was performed in SAS 94 M7 English for the PICO study and in R version 4.2.2 for the VASCO study. Statistical analyses of both studies were performed using R version 4.2.2.

## Results

### Study population

A total study population of 53,432 participants (VASCO: 45,413; PICO: 8,019) was included (**Table 1**). 90% of VASCO participants completed at least three SF-12 questionnaires (range: 1–5). For PICO participants, 50% participated in all three PICO rounds since new study participants were recruited in the third period (November 2021). VASCO participants were older than PICO participants (median 60 vs 53 years) and the age distribution differed between the two studies with the 50–70 year age groups being overrepresented in VASCO. Additionally, VASCO participants were more often female (62.9% vs 57.3%) and highly educated (56.9% vs 48.4%). The distributions of ethnicities (89.5% vs 89.2% Dutch) and having at least one comorbidity (33.0% vs 32.6%) were comparable between the two studies. The largest VASCO subgroup was 18–59 year-olds without a medical risk condition (N = 17,326), followed by 60–85 year-olds without a medical risk condition (N = 14,701), 60–85 year-olds with a medical risk condition (N = 9,133), and 18–59 year-olds with a medical risk condition (N = 4,253). For PICO, the respective group sizes were 4,097, 1,701, 1,472 and 749. The percentages of participants with missing covariate data on age, sex and comorbidities was limited (VASCO: 0.3%; PICO: 0.2%).

**Table 1:** Characteristics of all PICO and VASCO participants included in this study^a^.

	VASCO	PICO
	(n = 45,413)	(n = 8,019)
**Age (years)**		
**Median (IQR)**	60.0 (16)	53.0 (31)
**Age group (years) *[n (%)]***		
**18–24**	1,187 (2.6)	653 (8.1)
**25–29**	1,539 (3.4)	551 (6.9)
**30–34**	1,764 (3.9)	504 (6.3)
**35–39**	1,895 (4.2)	627 (7.8)
**40–44**	2,385 (5.3)	592 (7.4)
**45–49**	3,264 (7.2)	585 (7.3)
**50–54**	5,407 (11.9)	678 (8.5)
**55–59**	4,138 (9.1)	656 (8.2)
**60–64**	11,438 (25.2)	665 (8.3)
**65–69**	7,503 (16.5)	680 (8.5)
**70–74**	3,280 (7.2)	714 (8.9)
**75–79**	1,270 (2.8)	717 (8.9)
**80–85**	343 (0.8)	397 (5.0)
**Sex *[n (%)]***		
**Male**	16,824 (37.0)	3,422 (42.7)
**Female**	28,565 (62.9)	4,597 (57.3)
**Other**	24 (0.1)	0 (0.0)
**Educational level**^**b**^ ***[n (%)]***		
**Low**	6,294 (13.9)	1,636 (20.4)
**Intermediate**	13,043 (28.7)	2,488 (31.0)
**High**	25,820 (56.9)	3,878 (48.4)
**Other**	254 (0.6)	0 (0.0)
**Missing**	2 (0.0)	17 (0.2)
**Ethnicity *[n (%)]***		
**Dutch**	40,659 (89.5)	7,153 (89.2)
**Non-Dutch Western**	3,217 (7.1)	619 (7.7)
**Non-Western**	1,451 (3.2)	247 (3.1)
**Missing**	86 (0.2)	0 (0.0)
**Comorbidities *[n (%)]***		
**No**	30,438 (67.0)	5,406 (67.4)
**1**	10,882 (24.0)	1,808 (22.5)
**2**	3,076 (6.8)	558 (7.0)
**3 or more**	1,017 (2.2)	247 (3.1)
**Riskgroup *[n (%)]***		
**18–59 years with a medical risk condition** ^**c**^	4,253 (9.4)	749 (9.3)
**18–59 years without a medical risk condition** ^**c**^	17,326 (38.2)	4,097 (51.1)
**60–85 years with a medical risk condition** ^**c**^	9,133 (20.1)	1,472 (18.4)
**60–85 years without a medical risk condition** ^**c**^	14,701 (32.4)	1,701 (21.2)

^a^ Characteristics at moment of completion of first SF-12 questionnaire are shown.

^b^ Educational level was classified as low (no education or primary education), intermediate (secondary school or vocational training), or high (bachelor’s degree, university).

^c^ A medical risk condition was defined as having at least one of the following conditions: asthma, diabetes (type 1, type 2 and type unknown), asplenia, cardiovascular disease, immune deficiency, cancer (currently, treated and currently, untreated), liver disease, lung disease, neurological disease, kidney disease and organ or bone marrow transplantation.

Each of the four selected time periods included different numbers of participants (**S1 File, Fig S1.1**). In period one only PICO participants were included (N = 5,507). Period two and three consisted of both VASCO and PICO participants with 15,902 (VASCO: 11,102; PICO: 4,800) and 15,166 (VASCO: 8,407; PICO: 6,759) participants respectively. Period four included only VASCO participants (N = 12,837). Participants in period one were the youngest (median age 54 years), were least often female (56.5%) and least often had a medical indication (25.9%) compared to period two, three and four (median age 62, 58 and 61 years; females 60.5%, 61.5% and 62.0%; medical indication 30.3%, 32.0% and 29.6%, respectively).

### HRQoL stratified by age group, sex and presence of medical risk condition

**Fig 2:**
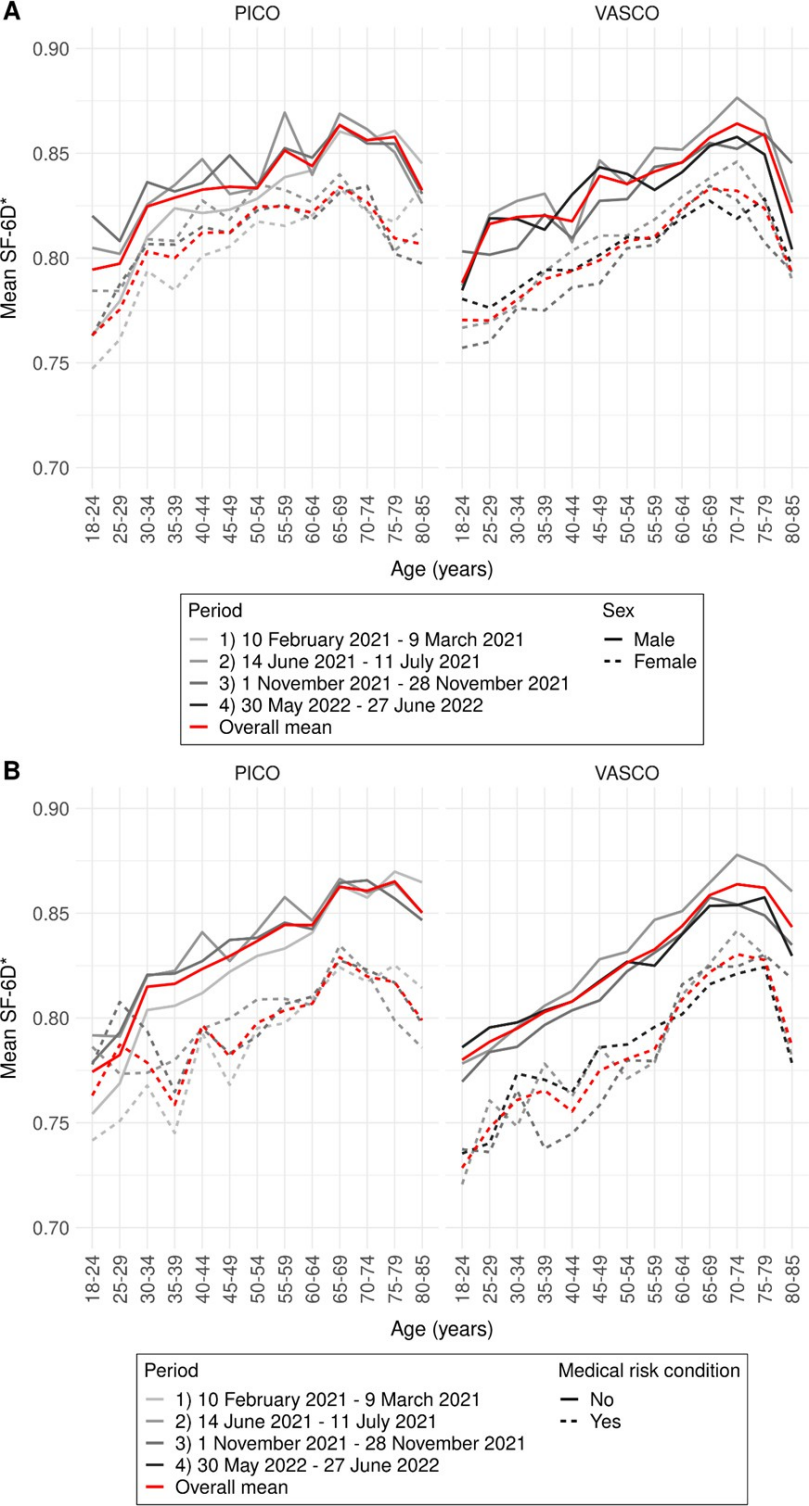
Health utility (SF-6D) in PICO and VASCO in four periods by age group and sex (A) and by age group and medical risk condition (B). Mean scores per study over the four periods are visualized by the red line.* Even though SF-6D scores can range from 0 to 1 (population average 0.8), y-axis ranges from 0.7 to 0.9 to better visualize differences.

**Fig 3:**
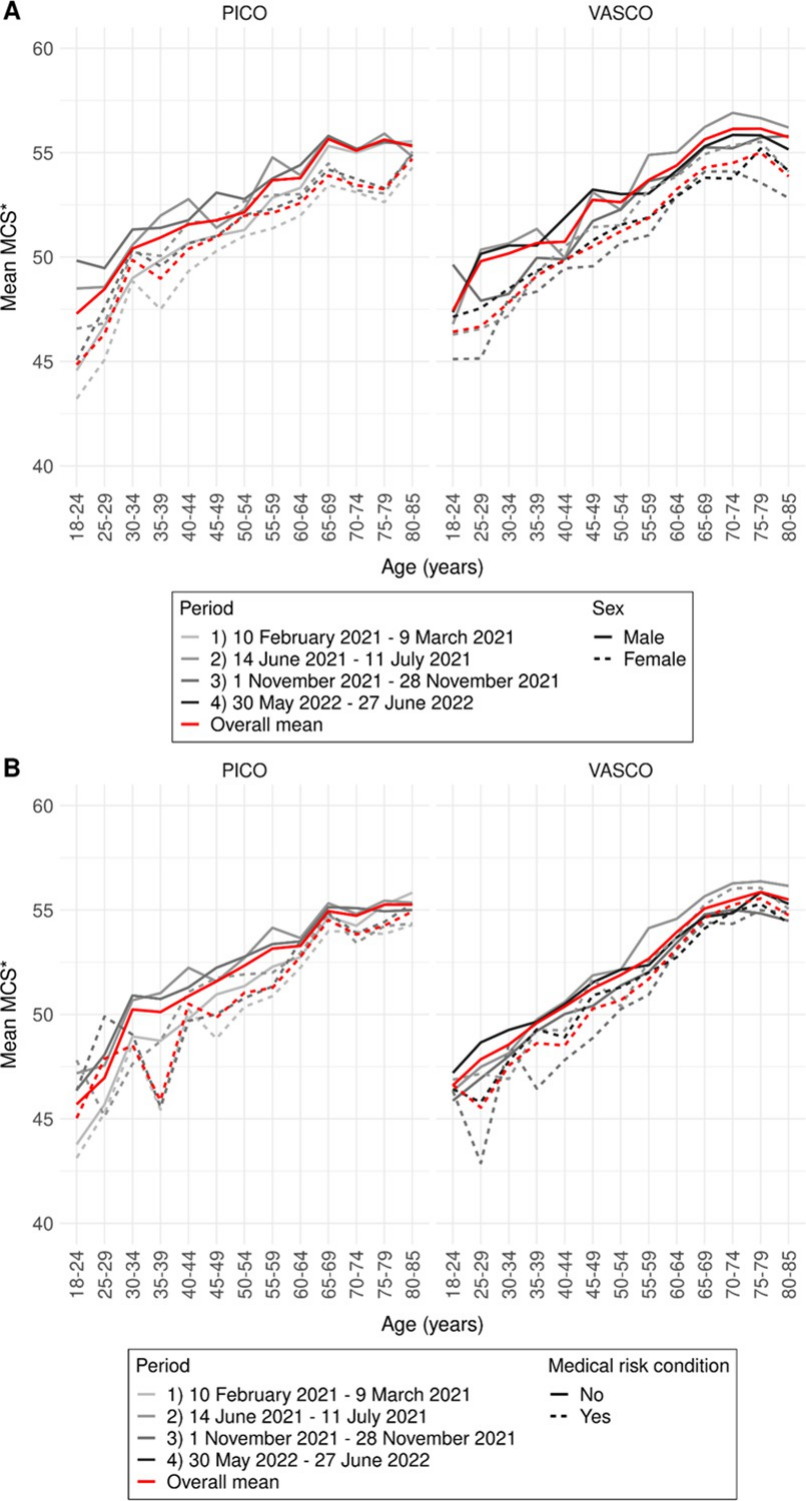
Mental health (MCS) in PICO and VASCO in four periods by age group and sex (A) and by age group and medical risk condition (B). Mean scores per study over the four periods are visualized by the red line.* Even though MCS scores can range from 0 to 100 (population average 50), y-axis ranges from 40 to 60 to better visualize differences.

**Fig 4:**
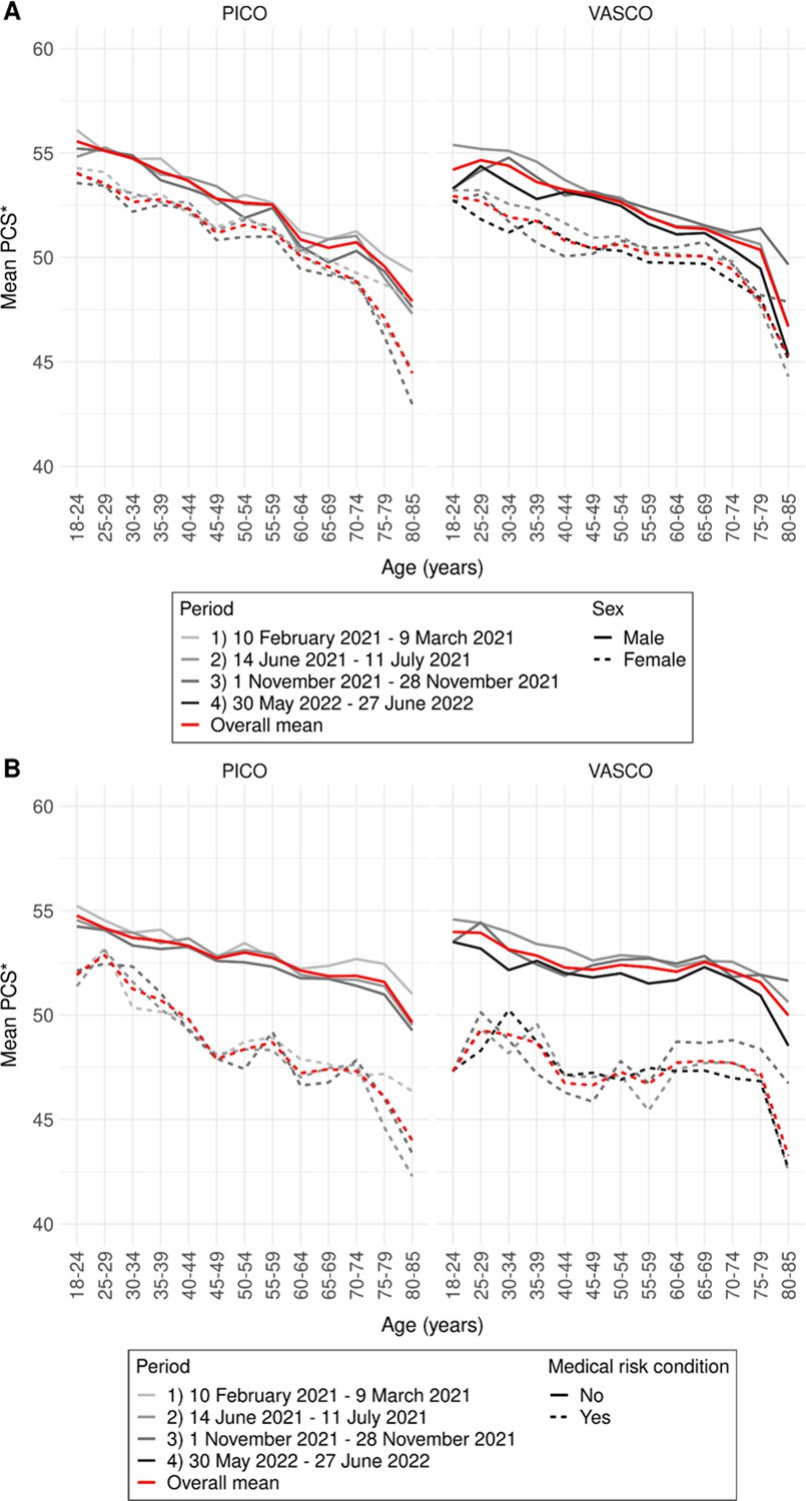
Physical health (PCS) in PICO and VASCO in four periods by age group and sex (A) and by age group and medical risk condition (B). Mean scores per study over the four periods are visualized by the red line.* Even though PCS scores can range from 0 to 100 (population average 50), y-axis ranges from 40 to 60 to better visualize differences.

VASCO and PICO data showed comparable trends in SF-6D utility scores (**Fig 2**), MCS (**Fig 3**), and PCS (**Fig 4**) by age group and sex, by age group and presence of a medical risk condition, and over periods. Therefore, we describe the average results over all periods of PICO participants only (all results are presented in **S1 File, Tables S1.1-S1.3**). Using Dutch general population weights or crude sum scores of SF-12 items associated with mental or physical health did not affect observed patterns (**S1 File, Figs S1.2-S1.7**). Weighting of the results to the general Dutch population only marginally influenced the results. These results were therefore disregarded in the final analysis. Separate mental and physical health items showed similar patterns by age group and are therefore not presented individually.

Mean SF-6D utility scores were lowest among 18–24 year-olds, increased with age up to 65–79 years, and declined thereafter (**Fig 2**). Between these age groups in PICO, mean male scores increased from 0.79 to 0.86 and then decreased to 0.83; mean scores of those without a medical risk condition increased from 0.77 to 0.87 and then decreased to 0.85 (**S1 File, Table S1.1A**). With significant differences observed for almost all age group (**S1 File, Table S1.1)**, female participants scored lower than males (**Fig 2A**) and participants with a medical risk condition had lower scores than those without (**Fig 2B**), with differences up to 0.05.

Mean MCS scores were also lowest among the youngest age groups up to 30 years (**Fig 3**). Scores increased with age up to 65–79 years and plateaued thereafter. Between these age groups in PICO, mean male scores increased from 47 to 56 and mean scores of participants without a medical risk condition increased from 46 to 55 (**S1 File, Table S1.2A**). On average, for the MCS again, females had lower scores than males with differences up to 2 (**Fig 3A**), and participants without a medical risk condition scored higher compared to those with a medical risk condition with differences up to 4 (**Fig 3B**). For the majority of age groups these differences between males and females and those with and without a medical risk condition were significant (**S1 File, Table S1.2**).

Contrary to SF-6D utility and MCS scores, mean PCS scores were highest in the youngest age groups up to 30 years (**Fig 4**). PCS decreased gradually with age up to 70–79 years and decreased sharply thereafter. Between these ages in PICO, mean male scores decreased from 56 to 48 and mean scores of participants without a medical risk condition from 55 to 50 (**S1 File, Table S1.3A**). Also, for PCS, mostly significantly lower scores were observed for females compared to males with differences up to 3 (**Fig 4A**).Larger, strongly significant differences (up to 6; **S1 File, Table S1.3**) were observed by presence of a medical risk condition, with lower scores for those with a medical risk condition (**Fig 4B**).

### HRQoL over time and correlation with stringency of restrictive measures

**Fig 5:**
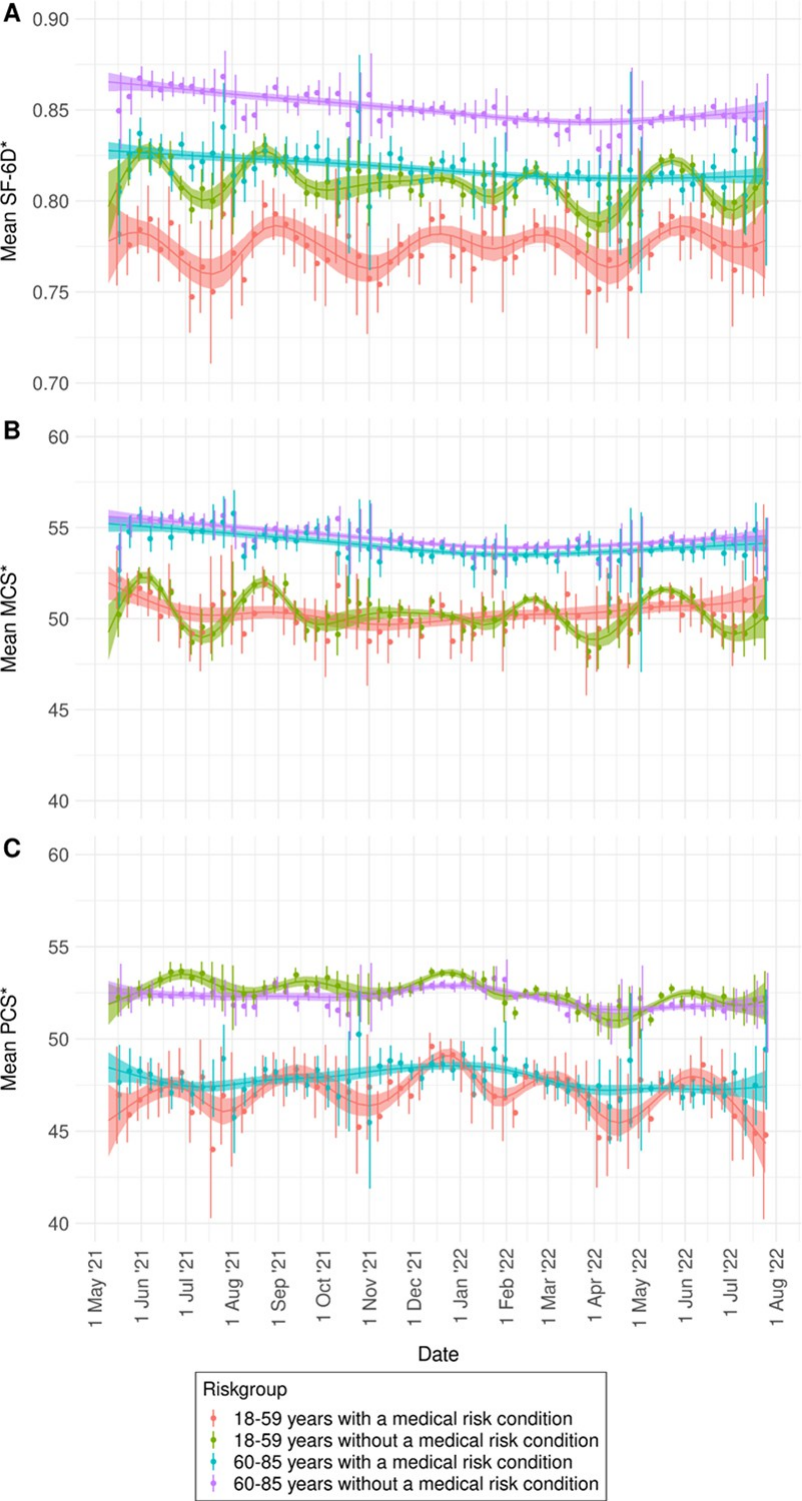
Weekly smoothed mean of (A) health utility (SF-6D), (B) mental health (MCS), and (C) physical health (PCS) by risk group in the VASCO cohort between 17-05-2021 and 31-07-2022. * Even though SF-6D and MCS/PCS scores can respectively range from 0 to 1 (population average 0.8) and 0 to 100 (population average 50), y-axes are shortened (respectively 0.7–0.9 and 40–60) to better visualize differences.

Mean weekly SF-6D utility, MCS and PCS scores in the VASCO cohort showed different patterns over time in the different risk groups (**Fig 5**). SF-6D utility score fluctuations over time were most pronounced among 18–59 year-olds, regardless of the presence of a medical risk condition. 60–85 year-olds showed more steady patterns of SF-6D utility scores over time, with patterns decreasing before stabilizing (those with a medical risk condition) or slightly increasing (those without a medical risk condition) from April 2022 onwards. For MCS, 18–59 year-olds without a medical risk condition showed strongest fluctuations over time, while 60–85 year-olds and 18–59 year-olds with a medical risk condition showed more steady patterns. Among 60–85 year-olds MCS patterns slowly decreased up until February 2022 and slightly increased thereafter. 18–59 year-olds with a medical risk condition showed an overall declining pattern up until November 2021, whereafter an increasing trend was observed. Lastly, PCS fluctuations over time were comparable between age groups, with 60–85 year-olds showing more steady patterns than those 18–59 years old. After a small peak in the second half of December 2021, decreasing patterns of PCS were observed in all groups between January and April 2022.

Statistically significant positive correlations between SF-6D utility scores and OxCGRT’s Stringency Index were found among those 18–59 years old without a medical risk condition (p = 0.001) and 60–85 year-olds with (p = 0.018) and without a medical risk condition (p<0.001), but correlation coefficients were small (0.16, 0.12 and 0.30, respectively) (**S1 File, Fig S1.8**). For MCS scores, statistically significant positive correlations with the Stringency Index were observed for those without a medical risk condition (18–59 years: p = 0.026; 60–85 years: p = 0.006), but correlation coefficients were again small (18–59 years: 0.11; 60–85 years: 0.14) (**S1 File, Fig S1.9**). PCS scores were statistically significantly, positively correlated with the Stringency Index among those 18–59 years old without a medical risk condition and 60–85 year-olds with and without a medical risk condition (p<0.001 in all groups) (**S1 File, Fig S1.10**). Correlation coefficients for PCS scores were small to moderate (respectively 0.40, 0.21 and 0.41 for each risk group).

In our sensitivity analysis with varying lag times of zero, one, three and four weeks no large differences were observed in the correlations between the Stringency Index and our outcomes (**S1 File, Table S1.4**). For SF-6D utility scores, statistically significant correlations were found for the same risk groups in all four lag time variations and correlation coefficients were comparable to those found in the two-week lag time analysis. Observed correlation coefficients for MCS scores were also comparable to those of the two-week lag time analysis. However, contrary to the two-week lag time analysis, MCS scores of those 18–59 years old without a medical risk condition were not statistically significantly correlated with the Stringency Index in the three- and four-week lag time analyses (p = 0.115 and p = 0.253 respectively). Additionally, in the four-week lag time analysis, 60–85 year-olds with a medical risk condition did show a statistically significant positive correlation between MCS scores and the Stringency Index (p = 0.014). For PCS scores, varying lag times also did not greatly influence correlation coefficients of the correlation with the Stringency Index. However, contrary to the two-week lag time analysis, a statistically significant positive correlation between PCS scores and the Stringency Index was observed among those 18–59 years old with a medical risk condition in the one-week lag time analysis (p = 0.016).

The sensitivity analysis comparing the pre-Omicron period and the Omicron period showed mostly non-significant correlations between the Stringency Index and our outcome measures, five statistically significant positive correlations and two negative ones: for MCS scores of 60–85 year-olds with (correlation coefficient: -0.24; p = 0.002) and without a medical risk condition (correlation coefficient: -0.25; p<0.001) during the Omicron period (**S1 File, Table S1.5**).

## Discussion

In this study we showed that during the second and third year of the COVID-19 pandemic, health utility and mental health scores declined substantially with decreasing age in the general Dutch population. Furthermore, health utility, mental and physical health scores were consistently lower among women as compared to men, and all scores were worse among participants with a medical risk condition compared to those without. Results were consistent between the two large study cohorts. Patterns of health utility, mental health and physical health showed small fluctuations over time (more pronounced among participants up to 60 years). Patterns were weakly but mostly positively significantly correlated with OxCGRT’s Stringency Index, unexpectedly suggesting that on average stricter measures were related with better health outcome scores.

Differences in SF-6D utility scores, mental health and physical health between age groups (up to 0.1, 10 and 9, respectively), sex (up to 0.05, 3.1 and 3.4, respectively) and those with and without a medical risk condition (up to 0.06, 4.2 and 6.7, respectively) exceed MCID values, thus indicating to be relevant determinants. Between periods, differences within specific groups were generally not larger than MCID values. In the lower and upper age categories, few differences were observed between periods that did exceed MCID values. However, these values were randomly observed between periods and were not consistent between cohorts. Differences over time within risk groups did not or only marginally (for those 18–59 years) exceed MCID. This suggests that time-influences on HRQoL are limited.

Sex-related differences in health utility, and mental and physical health have been described before [32, 33]. Multiple studies have reported that depressive symptoms might be more prevalent among women and therefore contribute to the difference in HRQoL [34, 35]. Besides psychological dimensions, physical aspects are reported to be of influence too [33, 34]. Other explanations reported are differences in coping mechanisms and character traits between males and females [35, 36]. Literature also substantiates the lower scores we observed among those with a medical risk condition. Worse quality of life has been reported among patients with diabetes or cancer as compared to the general population, usually explained by differences in physical functioning and well-being (e.g., pain and fatigue) or mental health (e.g., anxiety and depression) [37–39].

Age-related differences in HRQoL have also been observed by Statistics Netherlands [40] during the pandemic, in 2020 and 2021. In these data, comparable with our findings, a decreasing age pattern for physical health and increasing age pattern for mental health was observed. However, physical health was slightly higher among all age groups in our study population suggesting a more physically healthy population most likely due to healthy volunteer bias. Also, mental health appeared lower among younger age groups in our results (47 in males and 45 in females <25 years) as compared to CBS data (52 and 47, respectively). This might be the result of differences in the aggregation of age groups, since CBS data was available for 12–24 year-olds as compared to the 18–24 years group in our data. Furthermore, there might be influence of differences in study populations and time period of data collection. We do not expect the poor mental health we observed among young adults to be solely explained by infection rates as this was already found in February 2021 when absolute differences in seroprevalence between age groups were still limited [41].

Even though we were not able to draw a comparison with pre-pandemic data within our study population, such data on health utility, mental health and physical health are available in literature. In contrast to our results, pre-pandemic data in the Dutch population in 2013 from Statistics Netherlands [42] showed consistent MCS scores across age groups ranging between 50 and 54 in females and between 52 and 55 in males. While the MCS scores we observed in older age groups (ranging between 53–56 for those aged ≥ 65 years) were comparable with pre-pandemic scores (52–55), we found lower MCS scores in younger age groups (ranging between 45–50 for those aged <30 years) compared with pre-pandemic scores (50–54). Trends we observed in PCS scores over age and sex were comparable with pre-pandemic scores, although older participants scored slightly higher in our studies (among those aged ≥75 years, our scores were 47 for males and 45 for females, while respective 2013 CBS scores were 45 and 40). A study from the United Kingdom, analysing data collected during 2009–2010 of over 22,000 respondents from a representative sample of British citizens, reported SF-6D utility scores decreasing with age (0.86 to 0.70 for males and 0.83 to 0.64 for females between age groups 16–19 years and ≥85 years) [24]. This age pattern is opposite to our results, in which scores increased from 0.79 to 0.83 in males and from 0.76 to 0.81 in females between our youngest and oldest age groups. Although the CBS and UK study are designed to measure trends over time in similar populations, differences in study design and study population make direct comparison of the outcomes difficult and both CBS and UK data included 16 and 17 year-olds in their youngest age group. Nevertheless, we would like to stress that we present a population estimate which is consistently lower over the time of a year, in two independent cohorts, and therefore this observed reduction in HRQoL driven by poor mental health among younger age groups warrants further research and follow up. If only to assure that the HRQoL does improve in the post pandemic years for this age group.

Literature studying HRQoL pre-pandemically as well as during the pandemic, showed that the COVID-19 pandemic had an impact on HRQoL. The UK Household Longitudinal Study using the General Health Questionnaire-12 showed that psychosocial distress substantially increased in the UK following the COVID-19 pandemic, especially in women and younger people [43]. A repeated cross-sectional analysis comparing results before and during the pandemic in the US using the Kessler 6 Psychological Distress Scale found a marked increase in psychological distress among adults with greatest relative increase in poor mental health in young people [44].

In contrast to what might be expected and has been found in literature [45], mainly positive correlations between HRQoL and strictness of COVID-19 measures were observed. It could be hypothesised that more stringent COVID-19 restrictions might be related with better mental health if people feel that measures will protect them [45, 46]. Also, stricter measures could indicate that the government is engaged and capable of a response to adequately manage the threat of the pandemic [46]. Adaptation to circumstances [47] has also been reported and is a possible explanation why no strong negative relationship was found in our study in later stages of the pandemic. However, it is also likely that other demographical factors, like working status and household size, or other aspects of the pandemic have influenced the trends in HRQoL over time. For example, greater intensity of the pandemic, including number of SARS-CoV-2 infections and related deaths, has been found to be associated with worse mental health [45]. However, while the greatest increase in infection-induced seroprevalence in the Netherlands occurred between November 2021 (25%) and March 2022 (60%) [41], mental health did not show a continuing decrease during this period. We do see a decreasing trend in physical health from January to April 2022, which is during the first and second Omicron wave when many infections occurred, possibly indicating that SARS-CoV-2 infections led to a decrease in physical health on the short-term. Since these high infection rates were accompanied by a declining stringency of restrictive measures, this could explain our finding of positive correlations between physical health and the Stringency Index during the Omicron period.

This study has several strengths. First, we analysed data of two different cohort studies with large study populations. The observed consistency increases the robustness of our findings. In addition, separately weighting the results of the two cohorts to the general Dutch population did not influence our results, thereby showing comparability of the two cohorts and their representativeness of the general Dutch population. Finally, the VASCO study design provided continuous data on HRQoL over a long period of time during the pandemic in which different restrictive measures were active. This enabled us to estimate the correlation with restrictions over time.

Some limitations need to be discussed. A major limitation for interpretation of the differences between pandemic and pre-pandemic data is that pre-pandemic data was collected in different study populations in 2012 and 2013. We were unable to determine whether observed differences are the result of the pandemic or whether trends were already present before the start of the pandemic and were caused by other factors changing over time. Multiple international studies before the COVID-19 pandemic already reported an increase in mental health problems in young adults [48]. Possible causes are societal pressure, social media, environmental issues, and educational stressors [48–51]. Also, changes may be explained by greater awareness for mental health in general and decreasing stigma in recent years [48].

Secondly, the Stringency Index is a composite measure based on multiple different metrics. It is possible that some of these metrics, that were in place in different compositions in different phases of the pandemic, were more related with mental health than others (e.g., stay-at-home requirements vs public information campaigns). Future research could therefore focus on these metrics separately. Next to this, the Stringency Index does not include whether persons adhere to the measures. Also, it has an irregular behaviour, with massive increases or decreases in a matter of one day. This makes interpretation of the correlation between the Stringency Index and HRQoL difficult. As the selection of the four-week periods for investigation of HRQoL by age and sex was guided by PICO study round execution, during period two (14-06-2021–11-07-2021), quite some measures were lifted causing the OxCGRT Stringency Index to drop from 62 to 32 on June 26, 2021. This could be of influence on the questionnaires completed in the first half of period two compared to those completed in the second half of the period. Since timing of participation in this round is not equally distributed over age groups, sex and medical risk condition, interpretation of results are more difficult in this period. However, scores of period two by subgroup were similar to those of other periods, thus we believe that the overall impact of this problem on our results is limited.

In conclusion, on a Dutch population level, results using data from two cohort studies indicate that during the second and third year of the COVID-19 pandemic, HRQoL scores seemed to be more related with age, sex and presence of a medical risk condition than with the stringency of COVID-19 non-pharmaceutical measures. Contrary to pre-pandemic data in the Netherlands, health utility and mental health declined with decreasing age. This poor mental health and overall HRQoL among the younger age groups calls for monitoring in the post-pandemic years, and perhaps public health interventions.

## Supporting information

S1 File Additional figures and tables. (DOCX)

## Abbreviation list

BP: Bodily pain
CI: 95% confidence interval
COVID-19: Coronavirus disease 2019
GH–: General Health
HRQoL: Health-related quality of life
MCID: Minimal clinically important difference
MCS: Mental health component score
MH: Mental health
OxCGRT: Oxford Coronavirus Government Response Tracker
PCS: Physical health component score
PF: Physical function
PICO: PIENTER Corona
RE: Role limitations due to emotional problems
RP: Role limitations due to physical health problems
SARS-CoV-2: Severe acute respiratory syndrome-coronavirus
SD: Standard deviation
SF: Social functioning
SF-12: 12-item Short-Form Health Survey
SF-6D: Short-Form Six Dimensions
VASCO: VAccination Study COrona
VT: Vitality
