# Supplementary material for: Health-related quality of life during the COVID-19 pandemic: The impact of restrictive measures using data from two Dutch population-based cohort studies
Source: PLoS One. 2024 Mar 18;19(3):e0300324. doi: 10.1371/journal.pone.0300324 (PMC10947685; doi:10.1371/journal.pone.0300324)
Supplement: S1 File — (DOCX) [file pone.0300324.s001.docx]

**S1 File – Additional figures and tables**

***Fig S1.1.*** *Number of questionnaires of each cohort included per week during the study period with four observed periods colour-indicated.*


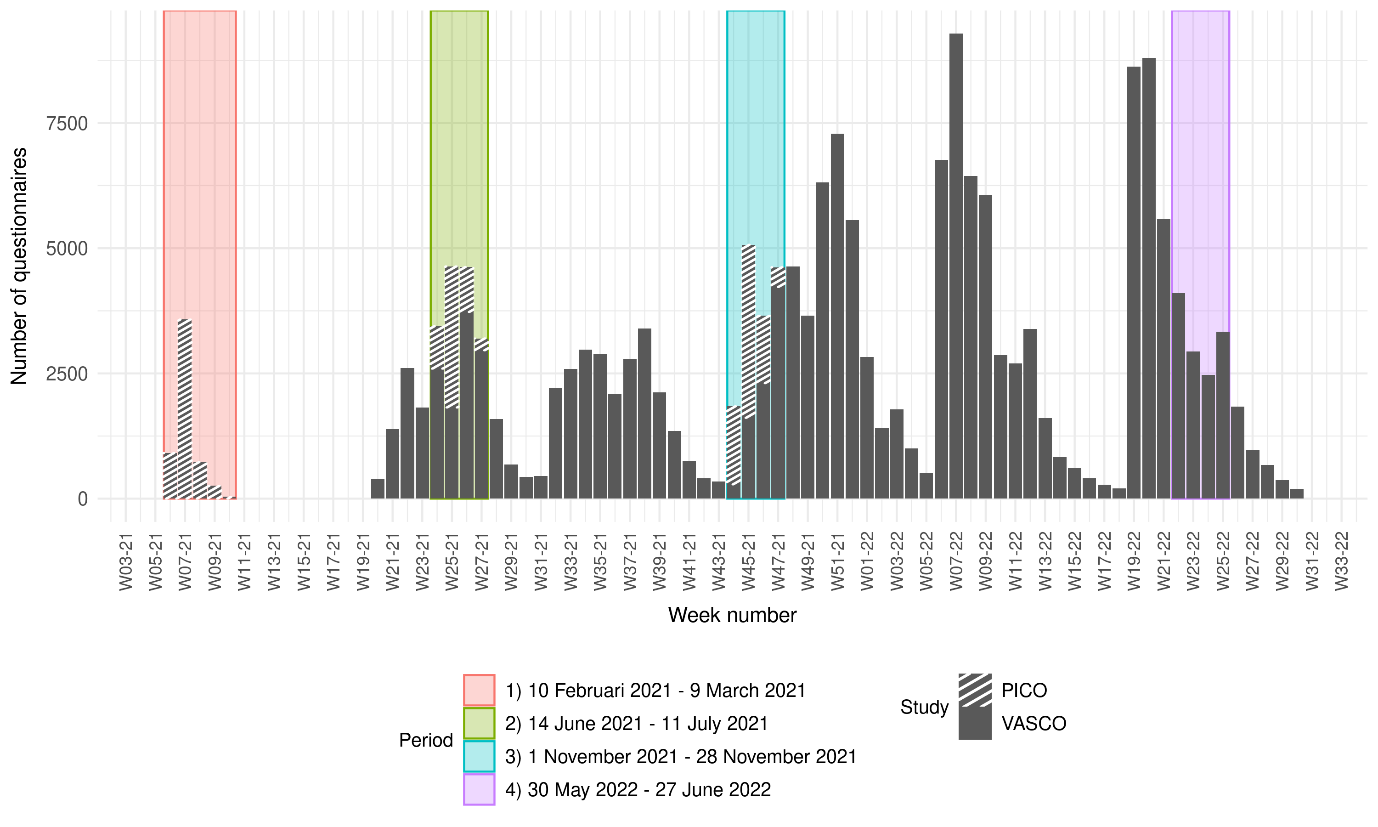


***Table S1.1.*** *Average SF-6D utility scores over all four periods by age group and sex and by age group and medical risk condition (mean (SD)) for PICO (A) and for VASCO (B).*

1. *PICO*

|  | Males | Females | P value | Without medical risk condition | With medical risk condition | P value |
| --- | --- | --- | --- | --- | --- | --- |
| 18-24 | 0.79 (0.11) | 0.76 (0.11) | <0.001 | 0.77 (0.11) | 0.76 (0.13) | 0.369 |
| 25-29 | 0.80 (0.10) | 0.78 (0.11) | 0.002 | 0.78 (0.11) | 0.79 (0.11) | 0.711 |
| 30-34 | 0.82 (0.10) | 0.80 (0.10) | 0.001 | 0.81 (0.10) | 0.78 (0.11) | <0.001 |
| 35-39 | 0.83 (0.10) | 0.80 (0.10) | <0.001 | 0.82 (0.10) | 0.76 (0.12) | <0.001 |
| 40-44 | 0.83 (0.10) | 0.81 (0.11) | 0.001 | 0.82 (0.10) | 0.80 (0.12) | 0.001 |
| 45-49 | 0.83 (0.12) | 0.81 (0.11) | 0.001 | 0.83 (0.11) | 0.78 (0.13) | <0.001 |
| 50-54 | 0.83 (0.10) | 0.82 (0.11) | 0.115 | 0.84 (0.10) | 0.80 (0.12) | <0.001 |
| 55-59 | 0.85 (0.10) | 0.82 (0.11) | <0.001 | 0.84 (0.10) | 0.80 (0.12) | <0.001 |
| 60-64 | 0.84 (0.12) | 0.82 (0.11) | <0.001 | 0.84 (0.10) | 0.81 (0.13) | <0.001 |
| 65-69 | 0.86 (0.10) | 0.83 (0.11) | <0.001 | 0.86 (0.10) | 0.83 (0.12) | <0.001 |
| 70-74 | 0.86 (0.10) | 0.83 (0.11) | <0.001 | 0.86 (0.10) | 0.82 (0.11) | <0.001 |
| 75-79 | 0.86 (0.11) | 0.81 (0.12) | <0.001 | 0.87 (0.10) | 0.82 (0.13) | <0.001 |
| 80-85 | 0.83 (0.11) | 0.81 (0.12) | 0.006 | 0.85 (0.11) | 0.80 (0.12) | <0.001 |

1. *VASCO*

|  | Males | Females | P value | Without medical risk condition | With medical risk condition | P value |
| --- | --- | --- | --- | --- | --- | --- |
| 18-24 | 0.79 (0.12) | 0.77 (0.11) | 0.067 | 0.78 (0.11) | 0.73 (0.12) | <0.001 |
| 25-29 | 0.82 (0.10) | 0.77 (0.11) | <0.001 | 0.79 (0.11) | 0.75 (0.13) | <0.001 |
| 30-34 | 0.82 (0.11) | 0.78 (0.12) | <0.001 | 0.79 (0.11) | 0.76 (0.12) | <0.001 |
| 35-39 | 0.82 (0.10) | 0.79 (0.11) | <0.001 | 0.80 (0.11) | 0.77 (0.13) | <0.001 |
| 40-44 | 0.82 (0.11) | 0.79 (0.11) | <0.001 | 0.81 (0.11) | 0.76 (0.11) | <0.001 |
| 45-49 | 0.84 (0.10) | 0.80 (0.11) | <0.001 | 0.82 (0.10) | 0.77 (0.13) | <0.001 |
| 50-54 | 0.84 (0.11) | 0.81 (0.11) | <0.001 | 0.83 (0.10) | 0.78 (0.12) | <0.001 |
| 55-59 | 0.84 (0.11) | 0.81 (0.11) | <0.001 | 0.83 (0.10) | 0.79 (0.12) | <0.001 |
| 60-64 | 0.85 (0.11) | 0.82 (0.11) | <0.001 | 0.84 (0.10) | 0.81 (0.12) | <0.001 |
| 65-69 | 0.86 (0.09) | 0.83 (0.10) | <0.001 | 0.86 (0.09) | 0.82 (0.11) | <0.001 |
| 70-74 | 0.86 (0.10) | 0.83 (0.10) | <0.001 | 0.86 (0.09) | 0.83 (0.11) | <0.001 |
| 75-79 | 0.86 (0.10) | 0.82 (0.11) | <0.001 | 0.86 (0.10) | 0.83 (0.11) | <0.001 |
| 80-85 | 0.82 (0.12) | 0.79 (0.11) | 0.025 | 0.84 (0.10) | 0.79 (0.12) | <0.001 |

***Table S1.2.*** *Average MCS over all four periods by age group and sex and by age group and medical risk condition (mean (SD)) for PICO (A) and for VASCO (B).*

1. *PICO*

|  | Males | Females | P value | Without medical risk condition | With medical risk condition | P value |
| --- | --- | --- | --- | --- | --- | --- |
| 18-24 | 47.3 (10.1) | 44.8 (10.5) | <0.001 | 45.7 (10.4) | 45.0 (10.9) | 0.560 |
| 25-29 | 48.5 (9.0) | 46.3 (10.2) | 0.001 | 47.0 (9.8) | 47.9 (10.7) | 0.451 |
| 30-34 | 50.4 (8.3) | 49.9 (8.4) | 0.297 | 50.2 (8.1) | 48.5 (10.6) | 0.050 |
| 35-39 | 50.9 (8.7) | 49.0 (9.1) | <0.001 | 50.1 (8.6) | 45.9 (11.0) | <0.001 |
| 40-44 | 51.6 (7.7) | 50.4 (8.8) | 0.015 | 50.9 (8.3) | 50.5 (8.8) | 0.583 |
| 45-49 | 51.8 (8.9) | 50.9 (8.7) | 0.115 | 51.6 (8.4) | 49.8 (9.9) | 0.005 |
| 50-54 | 52.1 (8.4) | 52.0 (7.8) | 0.710 | 52.3 (7.9) | 51.0 (8.5) | 0.008 |
| 55-59 | 53.7 (7.0) | 52.1 (8.2) | <0.001 | 53.2 (7.2) | 51.3 (9.2) | <0.001 |
| 60-64 | 53.8 (7.9) | 52.6 (7.4) | 0.002 | 53.3 (7.5) | 52.8 (7.8) | 0.190 |
| 65-69 | 55.7 (5.8) | 53.9 (7.1) | <0.001 | 54.9 (6.1) | 54.5 (7.1) | 0.189 |
| 70-74 | 55.1 (6.1) | 53.4 (7.3) | <0.001 | 54.7 (6.7) | 53.8 (6.8) | 0.006 |
| 75-79 | 55.6 (6.1) | 53.3 (7.6) | <0.001 | 55.3 (6.0) | 54.3 (7.4) | 0.007 |
| 80-85 | 55.3 (6.1) | 54.7 (6.4) | 0.227 | 55.3 (5.9) | 54.9 (6.4) | 0.498 |

1. *VASCO*

|  | Males | Females | P value | Without medical risk condition | With medical risk condition | P value |
| --- | --- | --- | --- | --- | --- | --- |
| 18-24 | 47.4 (10.7) | 46.4 (10.1) | 0.256 | 46.6 (10.3) | 46.6 (10.3) | 0.980 |
| 25-29 | 49.8 (9.5) | 46.7 (10.6) | <0.001 | 47.9 (10.2) | 45.5 (11.5) | 0.008 |
| 30-34 | 50.2 (9.5) | 47.8 (10.4) | <0.001 | 48.6 (10.2) | 47.5 (10.7) | 0.201 |
| 35-39 | 50.7 (8.9) | 49.1 (9.4) | 0.006 | 49.6 (9.1) | 48.6 (10.4) | 0.161 |
| 40-44 | 50.7 (9.4) | 49.9 (9.1) | 0.100 | 50.4 (8.9) | 48.5 (10.2) | 0.003 |
| 45-49 | 52.7 (8.1) | 50.5 (8.8) | <0.001 | 51.2 (8.5) | 50.3 (9.3) | 0.047 |
| 50-54 | 52.6 (8.0) | 51.2 (8.6) | <0.001 | 51.9 (8.2) | 50.7 (9.1) | 0.001 |
| 55-59 | 53.7 (7.5) | 51.9 (8.3) | <0.001 | 52.7 (7.9) | 51.7 (8.8) | 0.005 |
| 60-64 | 54.4 (6.9) | 53.3 (7.3) | <0.001 | 53.9 (6.9) | 53.2 (7.8) | <0.001 |
| 65-69 | 55.6 (5.6) | 54.3 (6.7) | <0.001 | 55.1 (5.8) | 54.6 (6.8) | 0.005 |
| 70-74 | 56.1 (5.6) | 54.5 (6.8) | <0.001 | 55.5 (5.9) | 55.2 (6.6) | 0.262 |
| 75-79 | 56.1 (5.2) | 55.0 (6.1) | <0.001 | 55.9 (5.3) | 55.5 (5.9) | 0.281 |
| 80-85 | 55.7 (6.7) | 53.9 (7.8) | 0.017 | 55.5 (6.5) | 54.7 (7.6) | 0.323 |

***Table S1.3.*** *Average PCS over all four periods by age group and sex and by age group and medical risk condition (mean (SD)) for PICO (A) and for VASCO (B).*

1. *PICO*

|  | Males | Females | P value | Without medical risk condition | With medical risk condition | P value |
| --- | --- | --- | --- | --- | --- | --- |
| 18-24 | 55.6 (4.2) | 54.0 (5.8) | <0.001 | 54.8 (5.1) | 51.9 (7.3) | <0.001 |
| 25-29 | 55.1 (4.1) | 53.6 (6.5) | <0.001 | 54.2 (5.9) | 52.9 (5.3) | 0.070 |
| 30-34 | 54.8 (4.1) | 52.7 (6.8) | <0.001 | 53.7 (5.6) | 51.3 (8.7) | <0.001 |
| 35-39 | 54.1 (4.8) | 52.8 (6.6) | <0.001 | 53.6 (5.7) | 50.7 (8.1) | <0.001 |
| 40-44 | 53.7 (5.6) | 52.3 (7.0) | <0.001 | 53.3 (6.0) | 49.8 (8.6) | <0.001 |
| 45-49 | 52.8 (6.8) | 51.2 (8.0) | <0.001 | 52.7 (6.5) | 47.9 (10.2) | <0.001 |
| 50-54 | 52.6 (6.6) | 51.6 (7.7) | 0.005 | 53.0 (6.2) | 48.4 (9.6) | <0.001 |
| 55-59 | 52.5 (6.3) | 51.3 (7.8) | 0.001 | 52.7 (6.3) | 48.7 (9.0) | <0.001 |
| 60-64 | 50.9 (7.6) | 50.1 (9.0) | 0.063 | 52.1 (6.6) | 47.2 (10.3) | <0.001 |
| 65-69 | 50.5 (7.8) | 49.5 (8.6) | 0.021 | 51.9 (6.3) | 47.4 (9.7) | <0.001 |
| 70-74 | 50.7 (7.5) | 48.8 (9.0) | <0.001 | 51.9 (6.4) | 47.3 (9.6) | <0.001 |
| 75-79 | 49.6 (8.2) | 47.1 (9.9) | <0.001 | 51.6 (6.9) | 46.1 (9.7) | <0.001 |
| 80-85 | 47.9 (9.0) | 44.5 (10.4) | <0.001 | 49.6 (7.8) | 44.0 (10.4) | <0.001 |

1. *VASCO*

|  | Males | Females | P value | Without medical risk condition | With medical risk condition | P value |
| --- | --- | --- | --- | --- | --- | --- |
| 18-24 | 54.2 (5.6) | 52.9 (7.3) | 0.038 | 54.0 (6.0) | 47.3 (10.4) | <0.001 |
| 25-29 | 54.7 (4.9) | 52.7 (7.6) | <0.001 | 53.9 (6.2) | 49.3 (9.8) | <0.001 |
| 30-34 | 54.4 (5.4) | 51.9 (8.0) | <0.001 | 53.1 (7.0) | 49.1 (9.3) | <0.001 |
| 35-39 | 53.6 (5.6) | 51.7 (7.8) | <0.001 | 52.8 (6.6) | 48.7 (10.0) | <0.001 |
| 40-44 | 53.2 (6.5) | 50.8 (8.5) | <0.001 | 52.3 (7.5) | 46.7 (9.8) | <0.001 |
| 45-49 | 53.0 (6.2) | 50.5 (8.7) | <0.001 | 52.2 (7.0) | 46.6 (10.7) | <0.001 |
| 50-54 | 52.6 (6.5) | 50.6 (8.5) | <0.001 | 52.4 (6.9) | 47.3 (10.2) | <0.001 |
| 55-59 | 52.0 (7.6) | 50.2 (9.1) | <0.001 | 52.3 (7.3) | 46.7 (10.6) | <0.001 |
| 60-64 | 51.4 (7.9) | 50.1 (8.6) | <0.001 | 52.1 (7.0) | 47.7 (9.8) | <0.001 |
| 65-69 | 51.4 (7.4) | 50.1 (8.7) | <0.001 | 52.5 (6.5) | 47.8 (9.5) | <0.001 |
| 70-74 | 50.8 (7.9) | 49.4 (8.7) | <0.001 | 52.1 (6.7) | 47.7 (9.4) | <0.001 |
| 75-79 | 50.4 (7.8) | 47.9 (9.4) | <0.001 | 51.6 (7.1) | 47.2 (9.3) | <0.001 |
| 80-85 | 46.7 (9.9) | 45.3 (9.9) | 0.199 | 50.0 (7.7) | 43.3 (10.4) | <0.001 |

***Fig S1.2.*** *Mental health (MCS) calculated using weights of the Dutch population in PICO and VASCO in four periods by age group and sex. Mean scores per study over the four periods are visualized by the red line.*

** Even though MCS scores can range from 0 to 100, y-axis ranges from 40 to 60 to better visualize differences.*


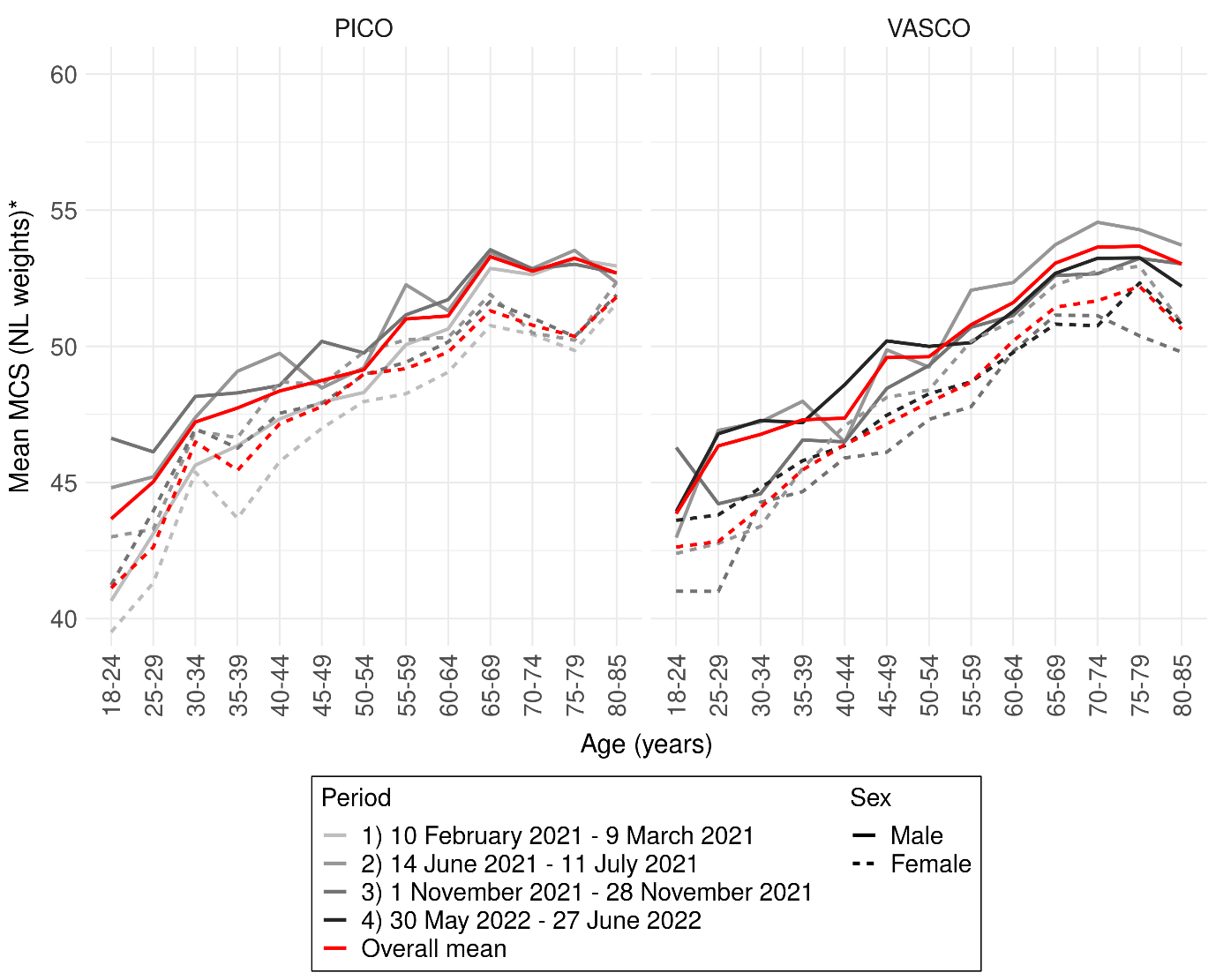


***Fig S1.3.*** *Physical health (PCS) calculated using weights of the Dutch population in PICO and VASCO in four periods by age group and sex. Mean scores per study over the four periods are visualized by the red line.*

** Even though PCS scores can range from 0 to 100, y-axis ranges from 40 to 60 to better visualize differences.*


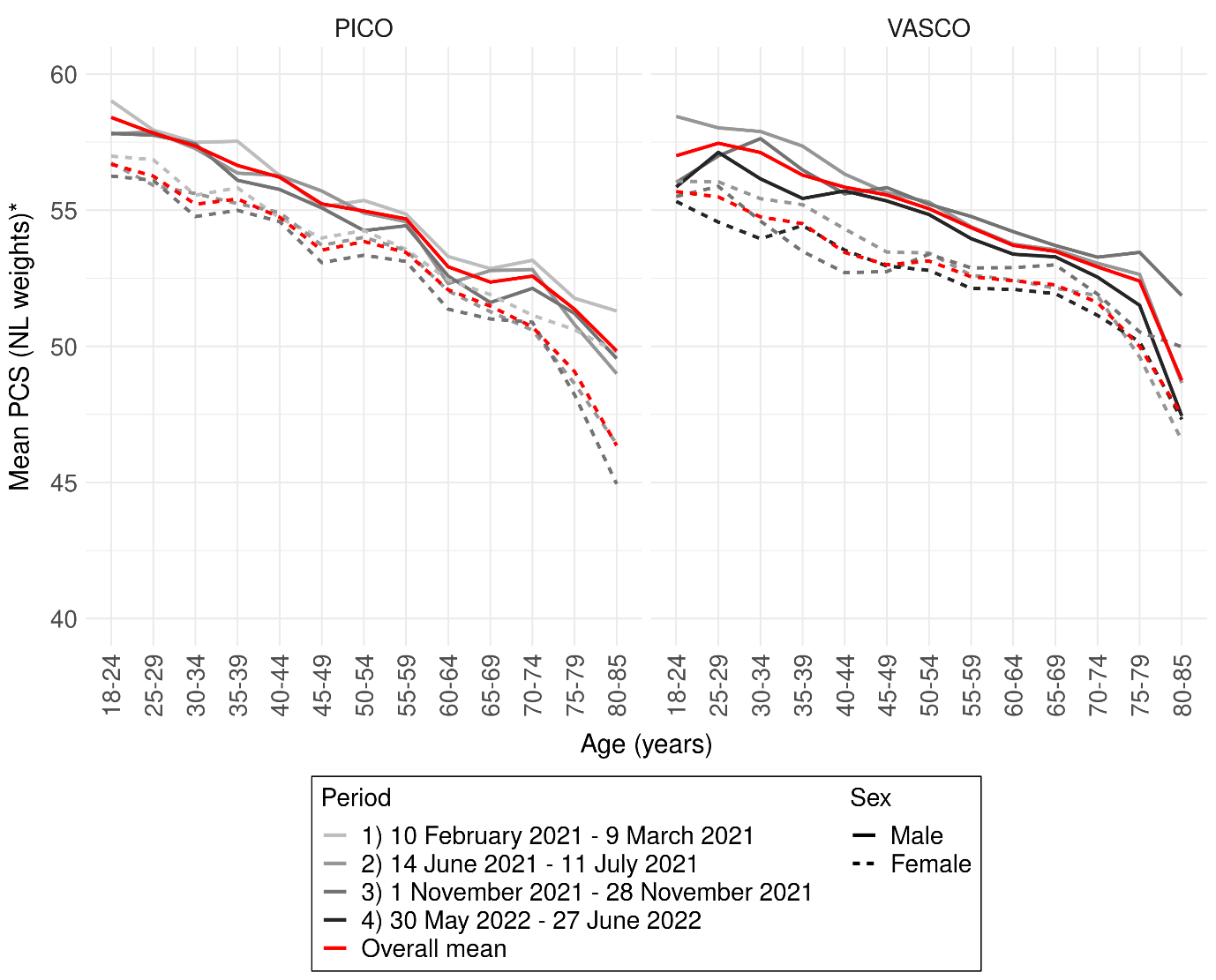


***Fig S1.4.*** *Weekly smoothed mean of (A) mental health (MCS), and (B) physical health (PCS) calculated using weights of the Dutch population by risk group in the VASCO cohort between 17-05-2021 and 31-07-2022.*

** Even though MCS and PCS scores can range from 0 to 100, y-axes are shortened (40-60) to better visualize differences.*


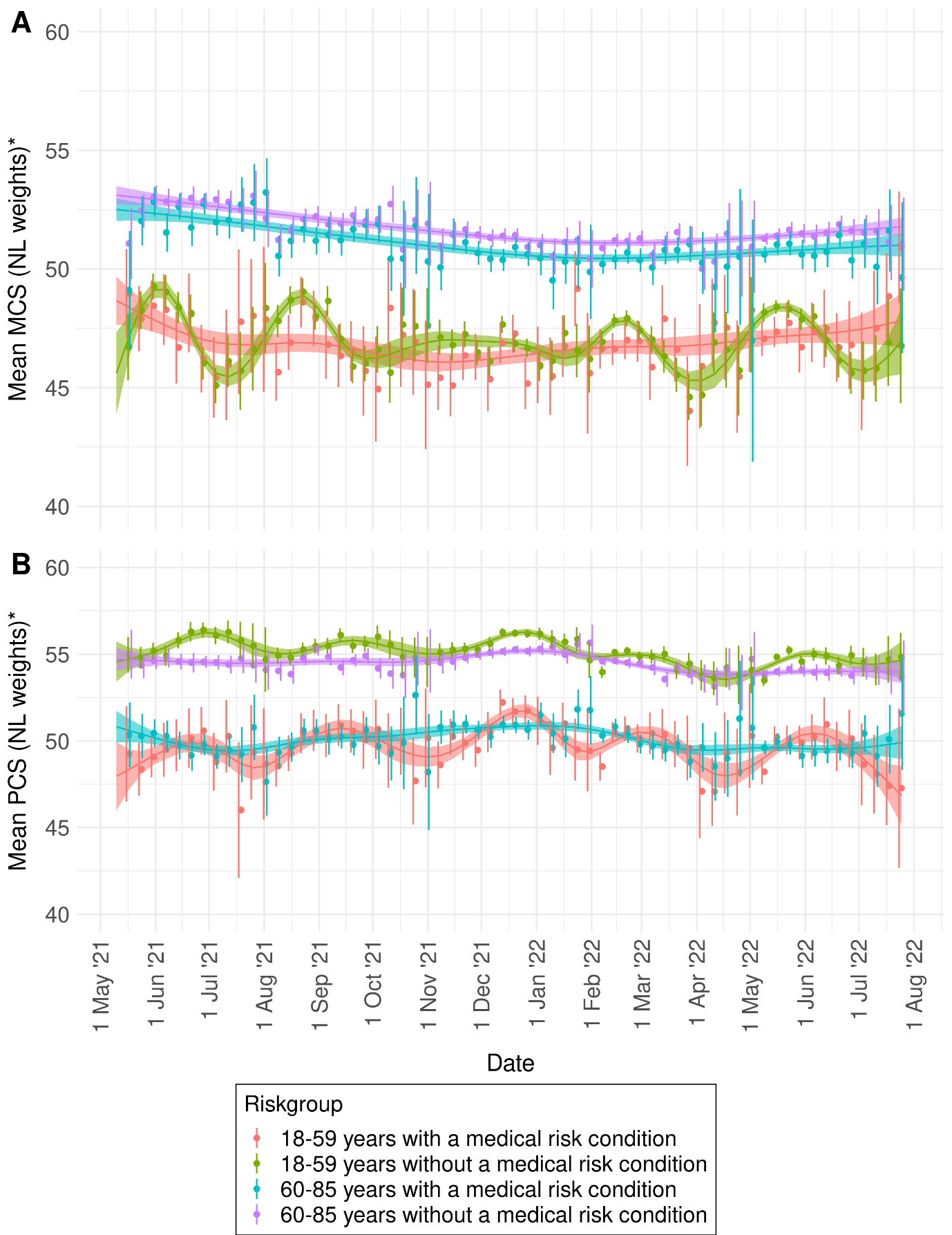


***Fig S1.5.*** *Mental health (raw sum score) in PICO and VASCO in four periods by age group and sex. Mean scores per study over the four periods are visualized by the red line.*

** Even though raw sum scores of mental health can range from 6 to 27, y-axis ranges from 17.5 to 25 to better visualize differences.*


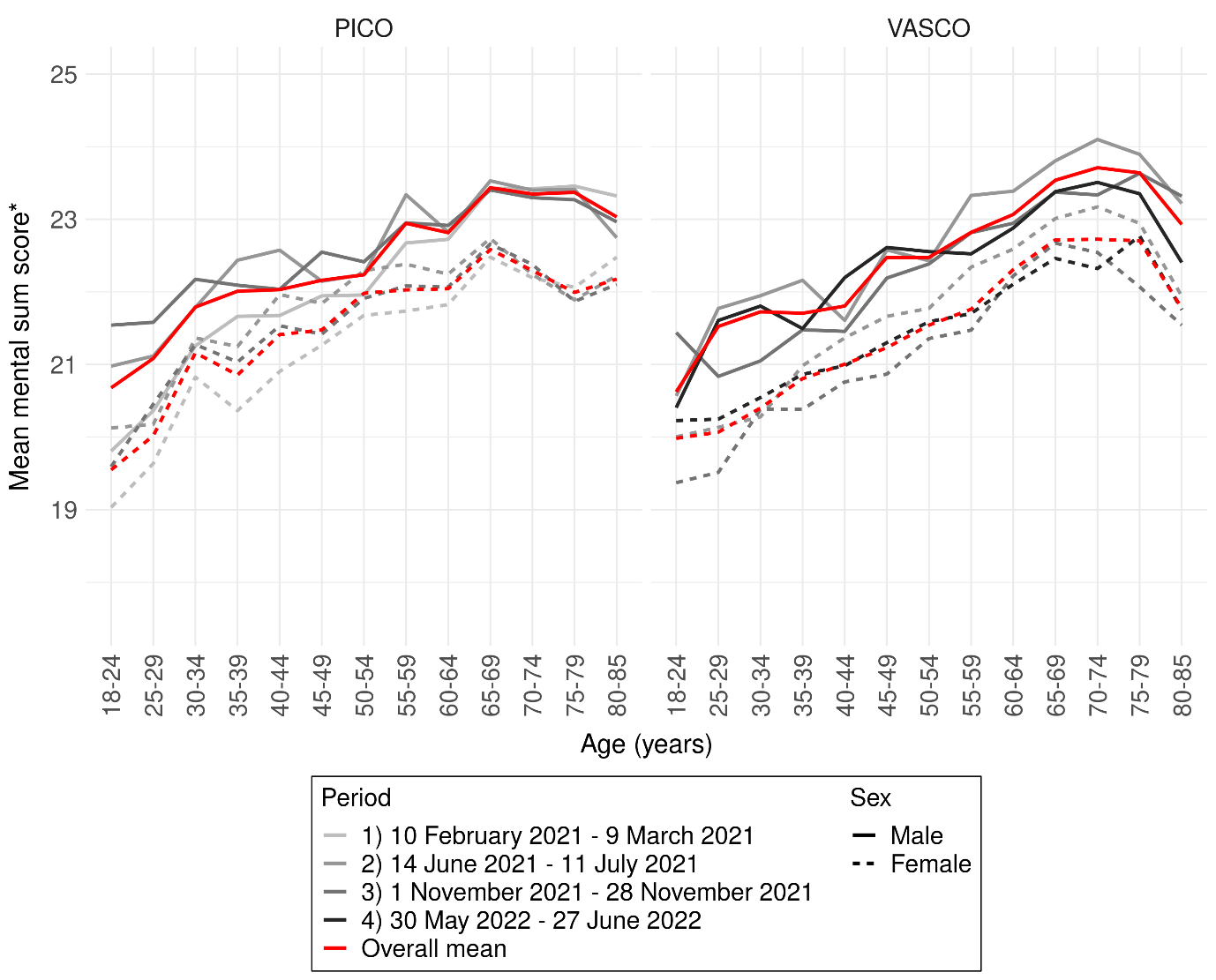


***Fig S1.6.*** *Physical health (raw sum score) in PICO and VASCO in four periods by age group and sex. Mean scores per study over the four periods are visualized by the red line.*

**
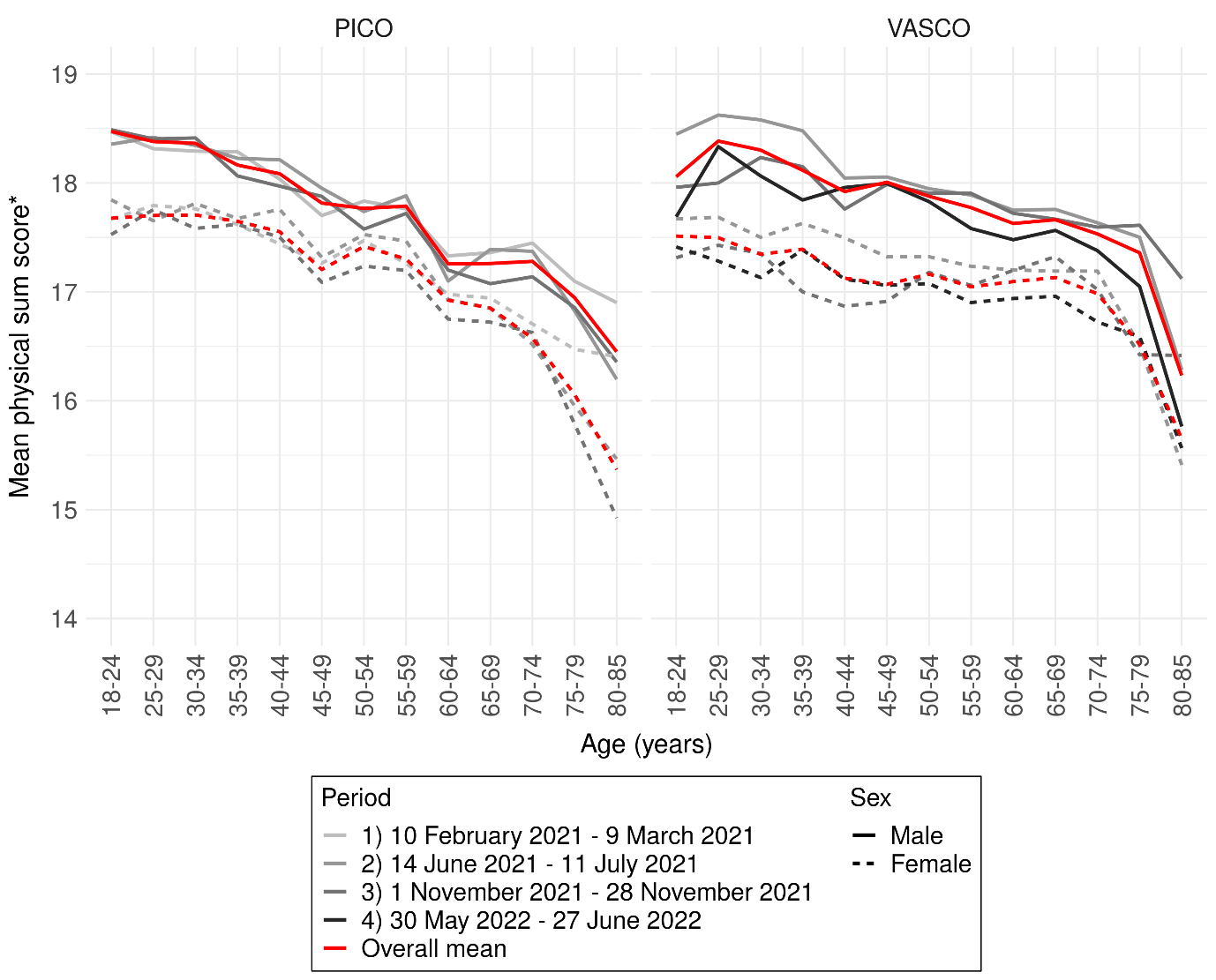
**** Even though raw sum scores of physical health can range from 6 to 20, y-axis ranges from 14 to 19 to better visualize differences.*

***Fig S1.7.*** *Weekly smoothed mean of (A) mental health, and (B) physical health (raw sum scores) by risk group in the VASCO cohort between 17-05-2021 and 31-07-2022.*

** Even though raw sum scores of mental health and physical health can respectively range from 6 to 27 and 6 to 20, y-axes are shortened (respectively 17.5-25 and 14-19) to better visualize differences.*

**
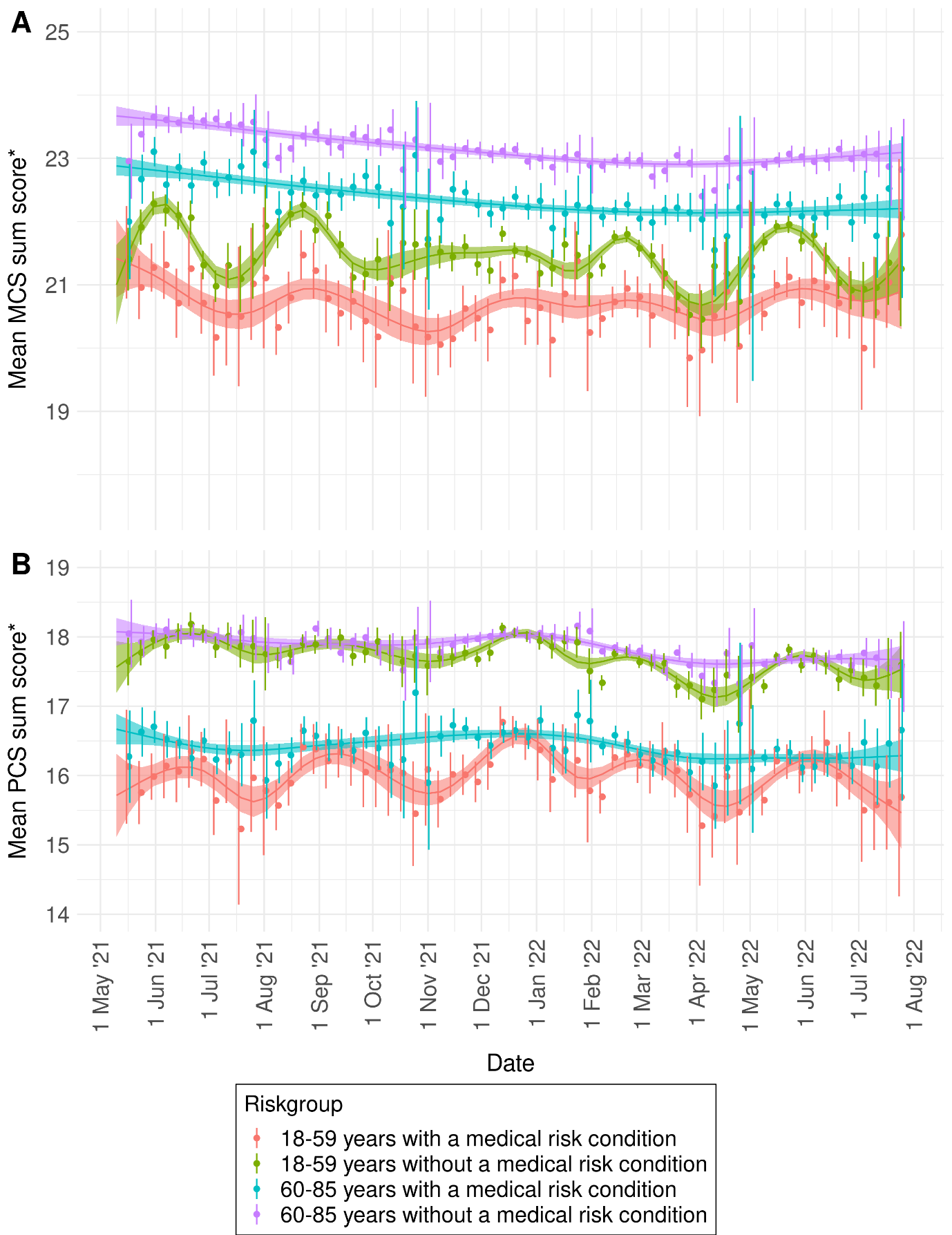
**


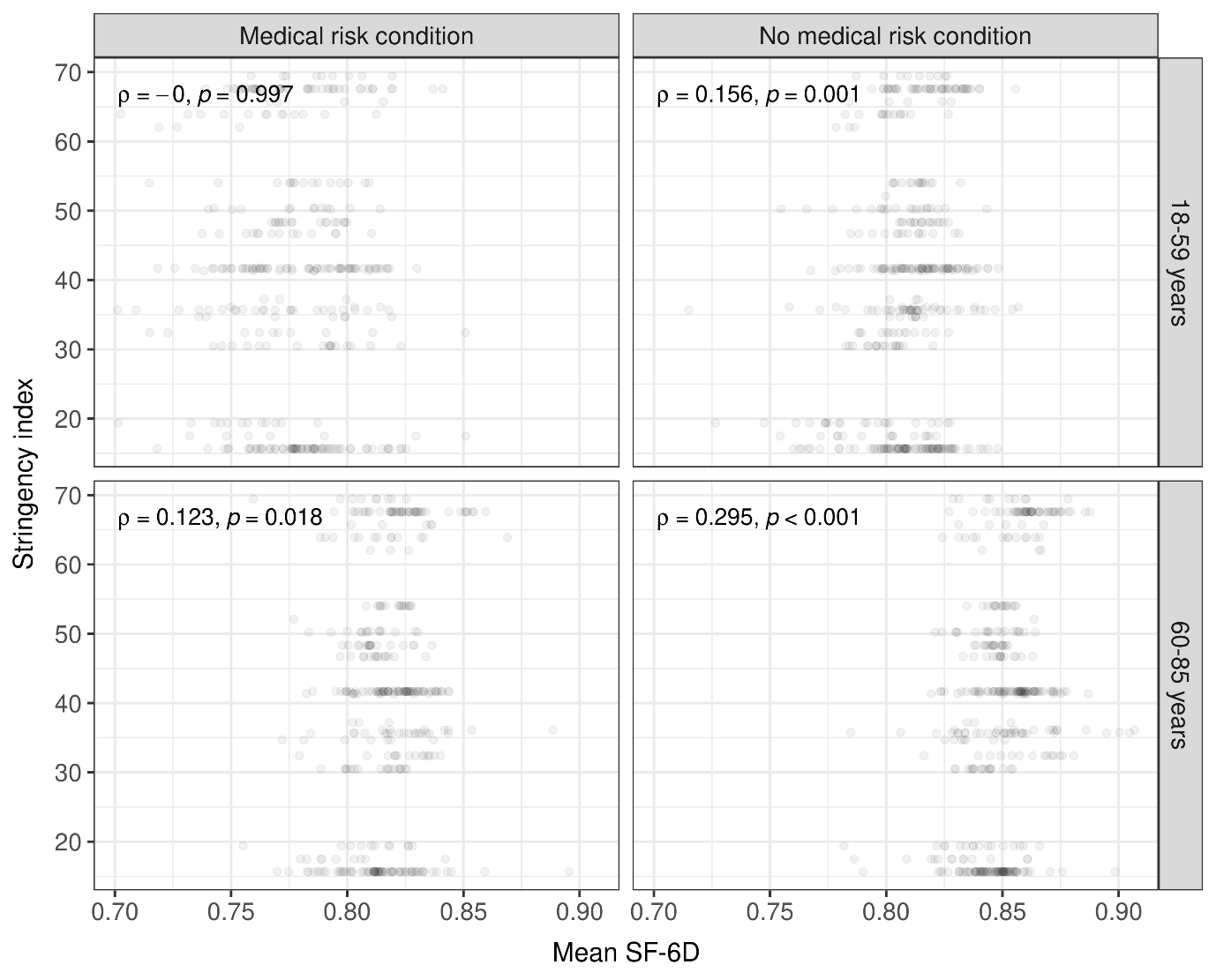
**Fig S1.8**. Correlation between OXCGRT’s Stringency Index and health utility (SF-6D) by risk group in VASCO.


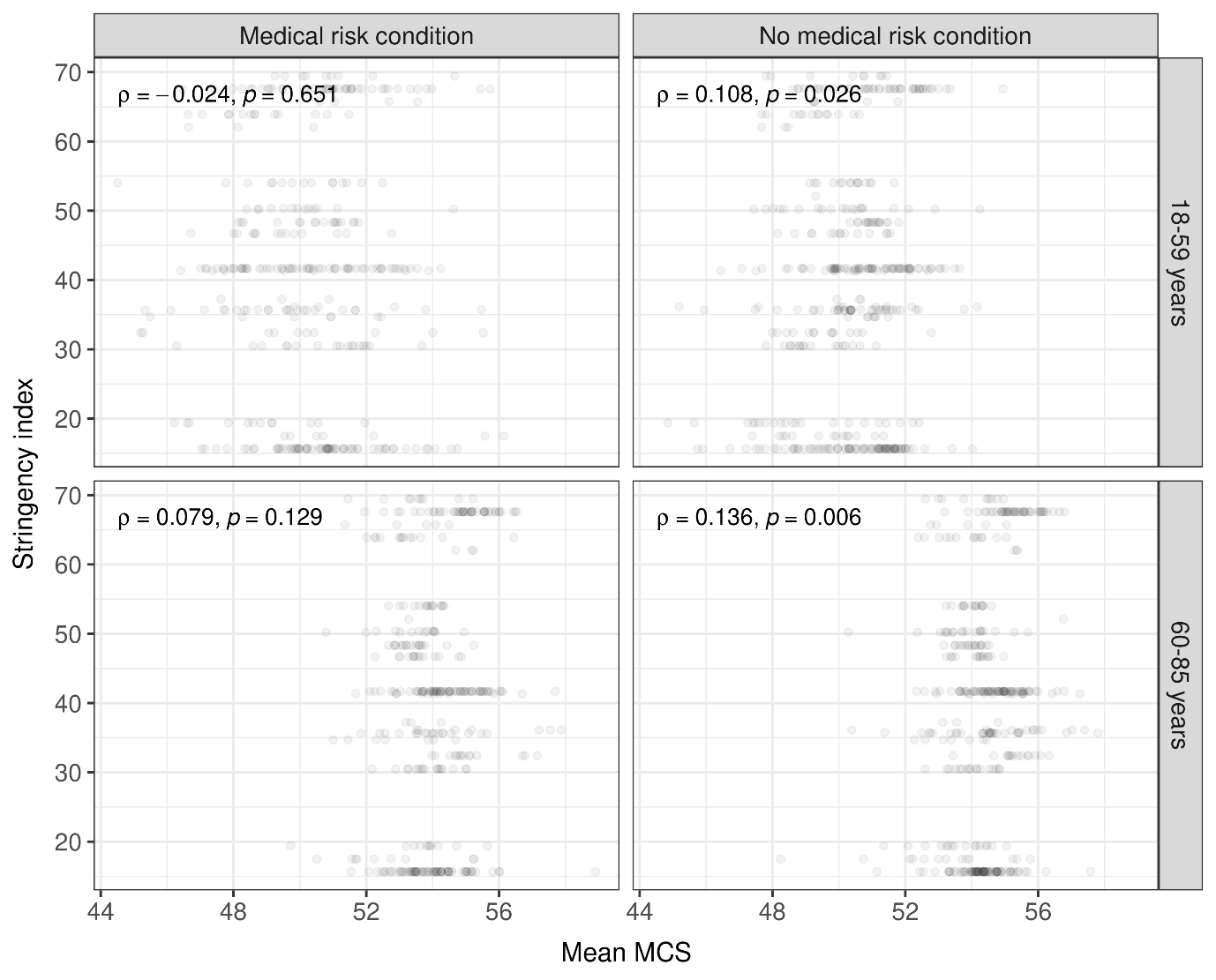
**Fig S1.9.** Correlation between OXCGRT’s Stringency Index and mental health (MCS) by risk group in VASCO.

**
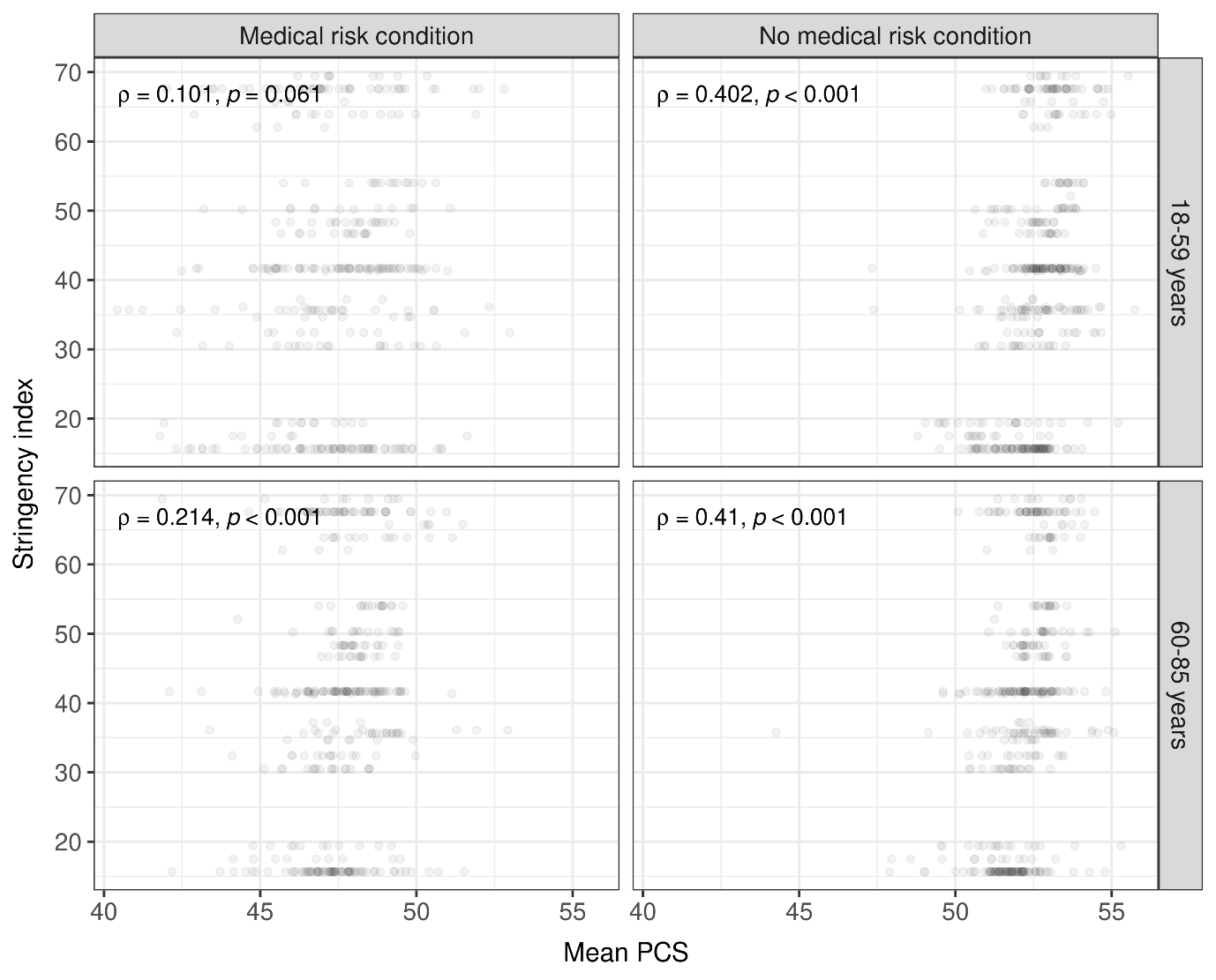
Fig S1.10**. Correlation between OXCGRT’s Stringency Index and physical health (PCS) by risk group in VASCO.

***Table S1.4.*** *Correlation between OXCGRT’s Stringency Index and health utility (SF-6D), mental health (MCS) and physical health (PCS) by risk group in VASCO with varying lag times of zero, one, three and four weeks.*

|  |  |  | 18-59 years with a medical risk condition | | 18-59 years without a medical risk condition | | 60-85 years with a medical risk condition | | 60-85 years without a medical risk condition | |
| --- | --- | --- | --- | --- | --- | --- | --- | --- | --- | --- |
|  |  |  | Correlation coefficient (ρ) | p-value | Correlation coefficient (ρ) | p-value | Correlation coefficient (ρ) | p-value | Correlation coefficient (ρ) | p-value |
| SF-6D | Number of weeks lag time | 0 | 0.007 | 0.9 | 0.16 | <0.001 | 0.156 | 0.003 | 0.257 | <0.001 |
|  |  | 1 | 0.037 | 0.49 | 0.176 | <0.001 | 0.158 | 0.002 | 0.294 | <0.001 |
|  |  | 3 | -0.008 | 0.888 | 0.135 | 0.006 | 0.123 | 0.019 | 0.299 | <0.001 |
|  |  | 4 | -0.01 | 0.847 | 0.105 | 0.031 | 0.132 | 0.012 | 0.277 | <0.001 |
| MCS | Number of weeks lag time | 0 | -0.041 | 0.446 | 0.122 | 0.012 | 0.048 | 0.36 | 0.1 | 0.045 |
|  |  | 1 | -0.019 | 0.721 | 0.129 | 0.008 | 0.084 | 0.107 | 0.124 | 0.012 |
|  |  | 3 | -0.026 | 0.63 | 0.077 | 0.115 | 0.078 | 0.136 | 0.159 | 0.001 |
|  |  | 4 | -0.01 | 0.854 | 0.056 | 0.253 | 0.127 | 0.014 | 0.17 | <0.001 |
| PCS | Number of weeks lag time | 0 | 0.103 | 0.055 | 0.37 | <0.001 | 0.294 | <0.001 | 0.408 | <0.001 |
|  |  | 1 | 0.129 | 0.016 | 0.398 | <0.001 | 0.262 | <0.001 | 0.423 | <0.001 |
|  |  | 3 | 0.092 | 0.086 | 0.385 | <0.001 | 0.197 | <0.001 | 0.387 | <0.001 |
|  |  | 4 | 0.071 | 0.186 | 0.358 | <0.001 | 0.13 | 0.012 | 0.342 | <0.001 |

***Table S1.5.*** *Correlation between OXCGRT’s Stringency Index and health utility (SF-6D), mental health (MCS) and physical health (PCS) by risk group in VASCO with two-week lag time in the pre-Omicron period (until 27 December 2021) and Omicron period (from 27 December 2021).*

|  |  | 18-59 years with a medical risk condition | | 18-59 years without a medical risk condition | | 60-85 years with a medical risk condition | | 60-85 years without a medical risk condition | |
| --- | --- | --- | --- | --- | --- | --- | --- | --- | --- |
|  |  | Correlation coefficient (ρ) | p-value | Correlation coefficient (ρ) | p-value | Correlation coefficient (ρ) | p-value | Correlation coefficient (ρ) | p-value |
| SF-6D | Pre-Omicron | 0.064 | 0.374 | 0.126 | 0.058 | -0.032 | 0.644 | 0.094 | 0.163 |
|  | Omicron | -0.044 | 0.59 | -0.093 | 0.193 | -0.106 | 0.181 | -0.01 | 0.892 |
| MCS | Pre-Omicron | 0.135 | 0.06 | 0.146 | 0.028 | 0.027 | 0.701 | 0.028 | 0.683 |
|  | Omicron | -0.116 | 0.155 | -0.12 | 0.093 | -0.242 | 0.002 | -0.25 | <0.001 |
| PCS | Pre-Omicron | 0.082 | 0.255 | 0.113 | 0.09 | 0.017 | 0.814 | 0.143 | 0.034 |
|  | Omicron | 0.036 | 0.658 | 0.215 | 0.002 | 0.233 | 0.003 | 0.397 | <0.001 |
